# Supplementary material for: Comparison of the Mental Health Impact of COVID-19 on Vulnerable and Non-Vulnerable Groups: A Systematic Review and Meta-Analysis of Observational Studies
Source: Int J Environ Res Public Health. 2021 Oct 15;18(20):10830. doi: 10.3390/ijerph182010830 (PMC8535316; doi:10.3390/ijerph182010830)
Supplement: Supplementary file 1 [file ijerph-18-10830-s001.zip › ijerph-1401124-supplementary.pdf]

Supplement 1. PRISMA 2020 Checklist

Section and Topic	Item #	Checklist item	Location where item is reported
**TITLE**			
Title	1	Identify the report as a systematic review.	Page 1
**ABSTRACT**			
Abstract	2	See the PRISMA 2020 for Abstracts checklist.	Page 1
**INTRODUCTION**			
Rationale	3	Describe the rationale for the review in the context of existing knowledge.	Page 1-2
Objectives	4	Provide an explicit statement of the objective(s) or question(s) the review addresses.	Page 2
**METHODS**			
Eligibility criteria	5	Specify the inclusion and exclusion criteria for the review and how studies were grouped for the syntheses.	Page 2-3
Information sources	6	Specify all databases, registers, websites, organisations, reference lists and other sources searched or consulted to identify studies. Specify the date when each source was last searched or consulted.	Page 2
Search strategy	7	Present the full search strategies for all databases, registers and websites, including any filters and limits used.	Supplementary 2
Selection process	8	Specify the methods used to decide whether a study met the inclusion criteria of the review, including how many reviewers screened each record and each report retrieved, whether they worked independently, and if applicable, details of automation tools used in the process.	Page 3
Data collection process	9	Specify the methods used to collect data from reports, including how many reviewers collected data from each report, whether they worked independently, any processes for obtaining or confirming data from study investigators, and if applicable, details of automation tools used in the process.	Page 3
Data items	10a	List and define all outcomes for which data were sought. Specify whether all results that were compatible with each outcome domain in each study were sought (e.g. for all measures, time points, analyses), and if not, the methods used to decide which results to collect.	Page 3
	10b	List and define all other variables for which data were sought (e.g. participant and intervention characteristics, funding sources). Describe any assumptions made about any missing or unclear information.	Page 3
Study risk of bias assessment	11	Specify the methods used to assess risk of bias in the included studies, including details of the tool(s) used, how many reviewers assessed each study and whether they worked independently, and if applicable, details of automation tools used in the process.	Page 4
Effect measures	12	Specify for each outcome the effect measure(s) (e.g. risk ratio, mean difference) used in the synthesis or presentation of	Page 4

Section and Topic	Item #	Checklist item	Location where item is reported
		results.	
Synthesis methods	13a	Describe the processes used to decide which studies were eligible for each synthesis (e.g. tabulating the study intervention characteristics and comparing against the planned groups for each synthesis (item #5)).	Page 4
	13b	Describe any methods required to prepare the data for presentation or synthesis, such as handling of missing summary statistics, or data conversions.	Page 4
	13c	Describe any methods used to tabulate or visually display results of individual studies and syntheses.	Page 4
	13d	Describe any methods used to synthesize results and provide a rationale for the choice(s). If meta-analysis was performed, describe the model(s), method(s) to identify the presence and extent of statistical heterogeneity, and software package(s) used.	Page 4
	13e	Describe any methods used to explore possible causes of heterogeneity among study results (e.g. subgroup analysis, meta-regression).	Page 4
	13f	Describe any sensitivity analyses conducted to assess robustness of the synthesized results.	NA
Reporting bias assessment	14	Describe any methods used to assess risk of bias due to missing results in a synthesis (arising from reporting biases).	Page 4
Certainty assessment	15	Describe any methods used to assess certainty (or confidence) in the body of evidence for an outcome.	NA
**RESULTS**			
Study selection	16a	Describe the results of the search and selection process, from the number of records identified in the search to the number of studies included in the review, ideally using a flow diagram.	Page 4
	16b	Cite studies that might appear to meet the inclusion criteria, but which were excluded, and explain why they were excluded.	Supplementary 3
Study characteristics	17	Cite each included study and present its characteristics.	Page 5-6
Risk of bias in studies	18	Present assessments of risk of bias for each included study.	Page 11
Results of individual studies	19	For all outcomes, present, for each study: (a) summary statistics for each group (where appropriate) and (b) an effect estimate and its precision (e.g. confidence/credible interval), ideally using structured tables or plots.	Page 14-17
Results of syntheses	20a	For each synthesis, briefly summarise the characteristics and risk of bias among contributing studies.	Page 14-17
	20b	Present results of all statistical syntheses conducted. If meta-analysis was done, present for each the summary estimate	Page 14-17

Section and Topic	Item #	Checklist item	Location where item is reported
		and its precision (e.g. confidence/credible interval) and measures of statistical heterogeneity. If comparing groups, describe the direction of the effect.	
	20c	Present results of all investigations of possible causes of heterogeneity among study results.	Page 14-17
	20d	Present results of all sensitivity analyses conducted to assess the robustness of the synthesized results.	NA
Reporting biases	21	Present assessments of risk of bias due to missing results (arising from reporting biases) for each synthesis assessed.	Page 117
Certainty of evidence	22	Present assessments of certainty (or confidence) in the body of evidence for each outcome assessed.	NA
**DISCUSSION**			
Discussion	23a	Provide a general interpretation of the results in the context of other evidence.	Page 17-18
	23b	Discuss any limitations of the evidence included in the review.	Page 20
	23c	Discuss any limitations of the review processes used.	Page 20
	23d	Discuss implications of the results for practice, policy, and future research.	Page 18-20
**OTHER INFORMATION**			
Registration and protocol	24a	Provide registration information for the review, including register name and registration number, or state that the review was not registered.	Page 2
	24b	Indicate where the review protocol can be accessed, or state that a protocol was not prepared.	Page 2
	24c	Describe and explain any amendments to information provided at registration or in the protocol.	Page 2
Support	25	Describe sources of financial or non-financial support for the review, and the role of the funders or sponsors in the review.	Page 21
Competing interests	26	Declare any competing interests of review authors.	Page 21
Availability of data, code and other materials	27	Report which of the following are publicly available and where they can be found: template data collection forms; data extracted from included studies; data used for all analyses; analytic code; any other materials used in the review.	Page 21

From: Page MJ, McKenzie JE, Bossuyt PM, Boutron I, Hoffmann TC, Mulrow CD, et al. The PRISMA 2020 statement: an updated guideline for reporting systematic reviews. BMJ 2021;372:n71. doi: 10.1136/bmj.n71

Supplement 2. Search terms used in each database

MEDLINE via PubMed

	Searches	Results
#1	"Vulnerable Populations"[MeSH] OR "Vulnerable Population*"[tiab] OR "Vulnerable Patient*"[tiab] OR vulnerable[tiab] OR Sensitive Population*[tiab] OR Sensitive Population Group*[tiab] OR Underserved Population*[tiab] OR Underserved Patient*[tiab] OR "Healthcare Disparities"[MeSH] OR disparities[tiab] OR disparity[tiab] OR Healthcare Disparity[tiab] OR Health Care Inequalities[tiab] OR Health Care Inequality[tiab] OR Healthcare Inequalities[tiab] OR Healthcare Inequality[tiab] OR Health Care Disparities[tiab] OR Health Care Disparity[tiab]OR disadvantage*[tiab] OR "Disabled persons"[MESH] OR Disability[tiab] OR Disabilities[tiab] OR Mental Disorders[MESH] OR "serious mental illness"[tiab] OR "Transients and Migrants"[MESH] OR Migrant*[tiab] OR "Emigrants and Immigrants"[MESH] OR Immigrant*[tiab] OR poverty[MESH] OR Poverty Areas[MESH] OR "low income"[tiab] OR Unemployment[MESH] OR Unemployment[tiab] OR Unemploy*[tiab] OR precarious[tiab] OR "Homeless Persons"[MESH] OR homeless[tiab] OR Aged[MeSH] OR elderly[tiab] OR"older adults"[tiab] OR "Multiple Chronic Conditions"[MESH] OR "chronic disease"[tiab] OR Single Parent[MESH] OR "single parent*"[tiab] OR Minority Groups[MESH] OR "ethnic minorities"[tiab] OR "racial minorities"[tiab] OR Refugees[MESH] OR refugee[tiab] OR Medically Uninsured[MESH] OR Uninsured[tiab] OR pregnant[MESH] OR pregnant[tiab] OR Minors [MESH] OR Child[MESH] OR child*[tiab]	6,845,997
#2	COVID-19[MeSH] OR COVID-19[tiab] OR COVID19[tiab] OR Coronavirus[tiab] OR Novel coronavirus[tiab] OR 2019-nCoV[tiab] OR SARS-CoV-2[tiab] OR SARS2[tiab] OR SARS-CoV[tiab] OR COVID 19[tiab] OR COVID-19 Virus Disease*[tiab] OR COVID 19 Virus Disease*[tiab] OR COVID-19 Virus Infection*[tiab] OR COVID 19 Virus Infection*[tiab] OR 2019-nCoV Infection*[tiab] OR 2019 nCoV Infection*[tiab] OR Coronavirus Disease-19[tiab] OR Coronavirus Disease 19[tiab] OR 2019 Novel Coronavirus Disease[tiab] OR 2019 Novel Coronavirus Infection[tiab] OR 2019-nCoV Disease*[tiab] OR 2019 nCoV Disease[tiab] OR COVID19[tiab] OR Coronavirus Disease 2019[tiab] OR SARS Coronavirus 2 Infection[tiab] OR SARS-CoV-2 Infection*[tiab] OR SARS CoV 2 Infection[tiab] OR COVID-19 Pandemic*[tiab] OR COVID 19 Pandemic[tiab]	118,916
#3	Mental health[MESH] OR "Mental health"[tiab] OR "Psychological health"[tiab] OR "Psychological impact*"[tiab] OR "Mental Hygiene"[tiab] OR Depression[MESH] OR Depression*[tiab] OR depress*[tiab] OR "Emotional Depression*"[tiab] OR Anxiety[MESH] OR Anxiety[tiab] OR Angst[tiab] OR Nervousness[tiab] OR Hypervigilance[tiab] OR Anxiousness[tiab] OR "Social Anxiety"[tiab] OR "Social Anxieties"[tiab] OR "Stress Disorders, Post-Traumatic"[MESH] OR PTSD[tiab] OR PTSS[tiab] OR "Post-traumatic stress disorder*"[tiab] OR "Post-Traumatic stress symptoms"[tiab] OR "Post-Traumatic Neuroses"[tiab] OR "Post Traumatic Neuroses"[tiab] OR PTSD[tiab] OR "Posttraumatic Neuroses"[tiab] OR "Post-Traumatic Stress Disorder*"[tiab] OR "Post Traumatic Stress Disorder*"[tiab] OR "Posttraumatic Stress Disorder*"[tiab] OR "Stress, Psychological"[MESH] OR Stress[tiab] OR "Psychological Stress*"[tiab] OR "Life Stress*"[tiab] OR "Psychologic Stress*"[tiab] OR "Psychological Stressor*"[tiab] OR Sleep[MESH] OR "Sleep Wake Disorders"[MESH] OR sleep*[tiab] OR insomnia*[tiab] OR wakeful*[tiab] OR dyssomn*[tiab] OR "Sleeping Habit*"[tiab] OR "Sleep Habit*"[tiab] OR "Sleep Wake Disorder*"[tiab] OR "Sleep Disorder*"[tiab]	1,734,050
#4	"animals"[MeSH Terms] NOT "humans"[MeSH Terms]	4,796,650
#5	#1 AND #2 AND #3 NOT #4	**3,861**

EMBASE via Elsevier

	Searches	Results
#1	'vulnerable population'/exp OR 'health care disparity'/exp OR 'mental disease'/exp OR 'disabled person'/exp OR 'migration'/exp OR 'poverty'/exp OR 'unemployment'/exp OR 'homeless person'/exp OR 'aged'/exp OR 'multiple chronic conditions'/exp OR 'single parent'/exp OR 'minority group'/exp OR 'refugee'/exp OR 'medically uninsured'/exp OR 'pregnant woman'/exp OR 'child'/exp OR 'minor person'/exp	8,025,493
#2	'vulnerable population*':ab,ti OR 'vulnerable patient*':ab,ti OR vulnerable:ab,ti OR disparities:ab,ti OR disparity:ab,ti OR disadvantage*:ab,ti OR disability:ab,ti OR 'disabilities serious mental illness':ab,ti OR migrant*:ab,ti OR immigrant*:ab,ti OR 'low income':ab,ti OR unemployment:ab,ti OR unemploy*:ab,ti OR precarious:ab,ti OR homeless:ab,ti OR elderly:ab,ti OR 'older adults':ab,ti OR 'chronic disease':ab,ti OR 'single parent*':ab,ti OR 'ethnic minorities':ab,ti OR 'racial minorities':ab,ti OR refugee:ab,ti OR uninsured:ab,ti OR pregnant:ab,ti OR child*:ab,ti	3,115,222
#3	#1 OR #2	8,983,421
	'coronavirus disease 2019'/exp	92,573
	coronavirus:ab,ti OR 'novel coronavirus':ab,ti OR '2019 ncov':ab,ti OR 'sars cov 2':ab,ti OR sars2:ab,ti OR 'sars cov':ab,ti OR 'covid 19':ab,ti OR 'covid-19 virus disease*':ab,ti OR 'covid 19 virus disease*':ab,ti OR 'covid-19 virus infection*':ab,ti OR 'covid 19 virus infection*':ab,ti OR '2019-ncov infection*':ab,ti OR '2019 ncov infection*':ab,ti OR 'coronavirus disease-19':ab,ti OR 'coronavirus disease 19':ab,ti OR '2019 novel coronavirus disease':ab,ti OR '2019 novel coronavirus infection':ab,ti OR '2019-ncov disease*':ab,ti OR '2019 ncov disease':ab,ti OR covid19:ab,ti OR 'coronavirus disease 2019':ab,ti OR 'sars coronavirus 2 infection':ab,ti OR 'sars-cov-2 infection*':ab,ti OR 'sars cov 2 infection':ab,ti OR 'covid-19 pandemic*':ab,ti OR 'covid 19 pandemic':ab,ti	114,539
	#4 OR #5	123,287
	'mental health'/exp OR 'depression'/exp OR 'anxiety'/exp OR 'posttraumatic stress disorder'/exp OR 'mental stress'/exp OR 'sleep'/exp OR 'sleep disorder'/exp	1,268,371
	'mental health':ab,ti OR 'psychological health':ab,ti OR 'psychological impact*':ab,ti OR 'mental hygiene':ab,ti OR depression*:ab,ti OR depress*:ab,ti OR 'emotional depression*':ab,ti OR anxiety:ab,ti OR angst:ab,ti OR nervousness:ab,ti OR hypervigilance:ab,ti OR anxiousness:ab,ti OR 'social anxiety':ab,ti OR 'social anxieties':ab,ti OR ptss:ab,ti OR 'post-traumatic stress symptoms':ab,ti OR 'post-traumatic neuroses':ab,ti OR 'post traumatic neuroses':ab,ti OR ptsd:ab,ti OR 'posttraumatic neuroses':ab,ti OR 'post-traumatic stress disorder*':ab,ti OR 'post traumatic stress disorder*':ab,ti OR 'posttraumatic stress disorder*':ab,ti OR stress:ab,ti OR 'psychological stress*':ab,ti OR 'life stress*':ab,ti OR 'psychologic stress*':ab,ti OR 'psychological stressor*':ab,ti OR sleep*:ab,ti OR insomnia*:ab,ti OR wakeful*:ab,ti OR dyssomn*:ab,ti OR 'sleeping habit*':ab,ti OR 'sleep habit*':ab,ti OR 'sleep wake disorder*':ab,ti OR 'sleep disorder*':ab,ti	2,071,874
	#7 OR #8	2,476,410
	'animal'/exp NOT 'human'/exp	5,579,871
	#3 AND #6 AND #9 NOT #10	**7,130**

Cochrane library

	Searches	Results
#1	MeSH descriptor: [Vulnerable Populations] explode all trees	308
#2	MeSH descriptor: [Healthcare Disparities] explode all trees	186
#3	MeSH descriptor: [Mental Disorders] explode all trees	74,488
#4	MeSH descriptor: [Disabled Persons] explode all trees	1,170
#5	MeSH descriptor: [Transients and Migrants] explode all trees	67
#6	MeSH descriptor: [Emigrants and Immigrants] explode all trees	175
#7	MeSH descriptor: [Poverty] explode all trees	1,756
#8	MeSH descriptor: [Poverty Areas] explode all trees	280
#9	MeSH descriptor: [Unemployment] explode all trees	100
#10	MeSH descriptor: [Homeless Persons] explode all trees	350
#11	MeSH descriptor: [Aged] explode all trees	209,996
#12	MeSH descriptor: [Multiple Chronic Conditions] explode all trees	32
#13	MeSH descriptor: [Single Parent] explode all trees	45
#14	MeSH descriptor: [Minority Groups] explode all trees	353
#15	MeSH descriptor: [Refugees] explode all trees	117
#16	MeSH descriptor: [Medically Uninsured] explode all trees	68
#17	MeSH descriptor: [Pregnant Women] explode all trees	290
#18	MeSH descriptor: [Child] explode all trees	56,688
#19	MeSH descriptor: [Minors] explode all trees	9
#20	(“Vulnerable Patient*” OR vulnerable OR “Sensitive Population*” OR “Sensitive Population Group*” OR “Underserved Population*” OR “Underserved Patient*” OR disparities OR disparity OR “Healthcare Disparity” OR “Health Care Inequalities” OR “Health Care Inequality” OR “Healthcare Inequalities” OR “Healthcare Inequality” OR “Health Care Disparities” OR “Health Care Disparity” OR disadvantage* OR Disability OR Disabilities OR “serious mental illness” OR Migrant* OR Immigrant* OR “low income” OR Unemployment OR Unemploy* OR precarious OR homeless OR elderly OR “older adults” OR "chronic disease" OR “single parent*” OR “ethnic minorities” OR “racial minorities” OR refugee OR Uninsured OR pregnant OR child*):ti,ab,kw	294,360
#21	#1 OR #2 OR #3 OR #4 OR #5 OR #6 OR #7 #8 OR #9 OR #10 OR #11 OR #12 OR #13 OR #14 OR #15 #16 OR #17 OR #18 OR #19 OR #20	515,103
#22	MeSH descriptor: [COVID-19] explode all trees	257
#23	(“COVID 19” OR COVID19 OR Coronavirus OR “Novel coronavirus” OR “2019 nCoV” OR “SARS CoV 2” OR SARS2 OR “SARS CoV” OR “COVID 19” OR “COVID 19” OR “Virus Disease*” OR “COVID 19” OR “Virus Disease*” OR “COVID 19” OR “Virus Infection*” OR “COVID 19” OR “Virus Infection*” OR “2019 nCoV Infection*” OR “2019 nCoV Infection*” OR “Coronavirus Disease 19” OR “Coronavirus Disease 19” OR “2019 Novel Coronavirus Disease” OR “2019 Novel Coronavirus Infection” OR “2019 nCoV Disease*” OR “2019 nCoV Disease” OR COVID19 OR “Coronavirus Disease 2019” OR “SARS Coronavirus 2 Infection” OR “SARS CoV 2 Infection*” OR “SARS CoV 2 Infection” OR “COVID 19 Pandemic*” OR “COVID 19 Pandemic”):ti,ab,kw	11,999
#24	#22 OR #23	11,999
#25	MeSH descriptor: [Mental Health] explode all trees	1,565

#26	MeSH descriptor: [Depression] explode all trees	12,583
#27	MeSH descriptor: [Anxiety] explode all trees	8,092
#28	MeSH descriptor: [Stress Disorders, Post-Traumatic] explode all trees	2,683
#29	MeSH descriptor: [Stress, Psychological] explode all trees	6,227
#30	MeSH descriptor: [Sleep] explode all trees	5,764
#31	MeSH descriptor: [Sleep Wake Disorders] explode all trees	8,318
#32	(“Mental health” OR “Psychological health” OR “Psychological impact*” OR “Mental Hygiene” OR Depression* OR depress* OR “Emotional Depression*” OR Anxiety OR Angst OR Nervousness OR Hypervigilance OR Anxiousness OR “Social Anxiety” OR “Social Anxieties” OR PTSD OR PTSS OR “Post-traumatic stress disorder*” OR “Post-Traumatic stress symptoms” OR “Post-Traumatic Neuroses” OR “Post Traumatic Neuroses” OR PTSD OR “Posttraumatic Neuroses” OR “Post-Traumatic Stress Disorder*” OR “Post Traumatic Stress Disorder*” OR “Posttraumatic Stress Disorder*” OR Stress OR “Psychological Stress*” OR “Life Stress*” OR “Psychologic Stress*” OR “Psychological Stressor*” OR sleep* OR insomnia* OR wakeful* OR dyssomn* OR “Sleeping Habit*” OR “Sleep Habit*” OR “Sleep Wake Disorder*” OR “Sleep Disorder*”):ti,ab,kw	202,391
#33	#25 OR #26 OR #27 OR #28 OR #29 OR #30 OR #31 OR #32	202,844
#34	MeSH descriptor: [Animals] explode all trees	599,447
#35	MeSH descriptor: [Humans] explode all trees	599,387
#36	#34 NOT #35	60
#37	#21 AND #24 AND #33 NOT #36	**406**

PsycINFO via EBSCO

	Searches	Results
#1	MA "Vulnerable Populations" OR “Healthcare Disparities” OR "Mental Disorders" OR Disadvantaged OR “Transients and Migrants” OR “Emigrants and Immigrants” OR poverty OR "Poverty Areas” OR Unemployment OR Homeless OR Aged OR "Chronic illness" OR "Single Parents" OR "Minority Groups" OR Refugees OR "Uninsured (Health Insurance)" OR pregnant OR Child OR Minors	629,760
#2	TI “Vulnerable Patient*” OR vulnerable OR Sensitive Population* OR Sensitive Population Group* OR Underserved Population* OR Underserved Patient* OR disparities OR disparity OR Healthcare Disparity OR Health Care Inequalities OR Health Care Inequality OR Healthcare Inequalities OR Healthcare Inequality OR Health Care Disparities OR Health Care Disparity OR disadvantage* OR Disability OR Disabilities OR “serious mental illness” OR Migrant* OR Immigrant* OR “low income” OR Unemployment OR Unemploy* OR precarious OR homeless OR elderly OR “older adults” OR "chronic disease" OR “single parent*” OR “ethnic minorities” OR “racial minorities” OR refugee OR Uninsured OR pregnant OR child*	471,180
#3	AB “Vulnerable Patient*” OR vulnerable OR Sensitive Population* OR Sensitive Population Group* OR Underserved Population* OR Underserved Patient* OR disparities OR disparity OR Healthcare Disparity OR Health Care Inequalities OR Health Care Inequality OR Healthcare Inequalities OR Healthcare Inequality OR Health Care Disparities OR Health Care Disparity OR disadvantage* OR Disability OR Disabilities OR “serious mental illness” OR Migrant* OR Immigrant* OR “low income” OR Unemployment OR Unemploy* OR precarious OR homeless OR elderly OR “older adults” OR "chronic disease" OR “single parent*” OR “ethnic minorities” OR “racial minorities” OR refugee OR Uninsured OR pregnant OR child*	975,611
#4	#1 OR #2 OR #3	1,048,739

#5	MA COVID-19	594
#6	TI COVID-19 OR COVID19 OR Coronavirus OR “Novel coronavirus” OR 2019-nCoV OR SARS-CoV-2 OR SARS2 OR SARS-CoV OR “COVID 19” OR “COVID-19 Virus Disease*” OR “COVID 19 Virus Disease*” OR “COVID-19 Virus Infection*” OR “COVID 19 Virus Infection*” OR “2019-nCoV Infection*” OR “2019 nCoV Infection*” OR “Coronavirus Disease-19” OR “Coronavirus Disease 19” OR “2019 Novel Coronavirus Disease” OR “2019 Novel Coronavirus Infection” OR “2019-nCoV Disease*” OR “2019 nCoV Disease” OR COVID19 OR “Coronavirus Disease 2019” OR “SARS Coronavirus 2 Infection” OR “SARS-CoV-2 Infection*” OR “SARS CoV 2 Infection” OR “COVID-19 Pandemic*” OR “COVID 19 Pandemic”	3,480
#7	AB COVID-19 OR COVID19 OR Coronavirus OR “Novel coronavirus” OR 2019-nCoV OR SARS-CoV-2 OR SARS2 OR SARS-CoV OR “COVID 19” OR “COVID-19 Virus Disease*” OR “COVID 19 Virus Disease*” OR “COVID-19 Virus Infection*” OR “COVID 19 Virus Infection*” OR “2019-nCoV Infection*” OR “2019 nCoV Infection*” OR “Coronavirus Disease-19” OR “Coronavirus Disease 19” OR “2019 Novel Coronavirus Disease” OR “2019 Novel Coronavirus Infection” OR “2019-nCoV Disease*” OR “2019 nCoV Disease” OR COVID19 OR “Coronavirus Disease 2019” OR “SARS Coronavirus 2 Infection” OR “SARS-CoV-2 Infection*” OR “SARS CoV 2 Infection” OR “COVID-19 Pandemic*” OR “COVID 19 Pandemic”	4,276
#8	#5 OR #6 OR #7	4,408
#9	MA "Mental health" OR "Depression (Emotion)" OR Anxiety OR “Posttraumatic Stress Disorder” OR Stress OR Sleep OR “Sleep Wake Disorders"	178,376
#10	TI “Mental health” OR “Psychological health” OR “Psychological impact*” OR “Mental Hygiene” OR Depression* OR depress* OR “Emotional Depression*” OR Anxiety OR Angst OR Nervousness OR Hypervigilance OR Anxiousness OR “Social Anxiety” OR “Social Anxieties” OR PTSD OR PTSS OR “Post-traumatic stress disorder*” OR “Post-Traumatic stress symptoms” OR “Post-Traumatic Neuroses” OR “Post Traumatic Neuroses” OR PTSD OR “Posttraumatic Neuroses” OR “Post-Traumatic Stress Disorder*” OR “Post Traumatic Stress Disorder*” OR “Posttraumatic Stress Disorder*” OR Stress OR “Psychological Stress*” OR “Life) Stress*” OR “Psychologic Stress*” OR “Psychological Stressor*” OR sleep* OR insomnia* OR wakeful* OR dyssomn* OR “Sleeping Habit*” OR “Sleep Habit*” OR “Sleep Wake Disorder*” OR “Sleep Disorder*”	337,290
#11	AB “Mental health” OR “Psychological health” OR “Psychological impact*” OR “Mental Hygiene” OR Depression* OR depress* OR “Emotional Depression*” OR Anxiety OR Angst OR Nervousness OR Hypervigilance OR Anxiousness OR “Social Anxiety” OR “Social Anxieties” OR PTSD OR PTSS OR “Post-traumatic stress disorder*” OR “Post-Traumatic stress symptoms” OR “Post-Traumatic Neuroses” OR “Post Traumatic Neuroses” OR PTSD OR “Posttraumatic Neuroses” OR “Post-Traumatic Stress Disorder*” OR “Post Traumatic Stress Disorder*” OR “Posttraumatic Stress Disorder*” OR Stress OR “Psychological Stress*” OR “Life) Stress*” OR “Psychologic Stress*” OR “Psychological Stressor*” OR sleep* OR insomnia* OR wakeful* OR dyssomn* OR “Sleeping Habit*” OR “Sleep Habit*” OR “Sleep Wake Disorder*” OR “Sleep Disorder*”	769,153
#12	#9 OR #10 OR #11	830,165
#13	MA animals NOT human	199,963
#14	#4 AND #8 AND #12 NOT #13	**648**

CINAHL via EBSCO

	Searches	Results
#1	MH ("Special Populations" OR “Healthcare Disparities” OR "Mental Disorders" OR Disabled OR “Transients and Migrants” OR Immigrants OR poverty OR "Indigent Persons" OR Poverty Areas OR Unemployment OR “Homeless Persons” OR Aged OR "Chronic Disease” OR "Single Parent" OR "Minority Groups" OR Refugees OR "Medically Uninsured" OR "Expectant Mothers" OR Child OR "Minors (Legal)") OR TI (“Vulnerable Patient*” OR vulnerable OR Sensitive Population* OR Sensitive Population Group* OR Underserved Population* OR Underserved Patient* OR disparities OR disparity OR Healthcare Disparity OR Health Care Inequalities OR Health Care Inequality OR Healthcare Inequalities OR Healthcare Inequality OR Health Care Disparities OR Health Care Disparity OR disadvantage* OR Disability OR Disabilities OR “serious mental illness” OR Migrant* OR Immigrant* OR “low income” OR Unemployment OR Unemploy* OR precarious OR homeless OR elderly OR “older adults” OR "chronic disease" OR “single parent*” OR “ethnic minorities” OR “racial minorities” OR refugee OR Uninsured OR pregnant OR child*) OR AB (“Vulnerable Patient*” OR vulnerable OR Sensitive Population* OR Sensitive Population Group* OR Underserved Population* OR Underserved Patient* OR disparities OR disparity OR Healthcare Disparity OR Health Care Inequalities OR Health Care Inequality OR Healthcare Inequalities OR Healthcare Inequality OR Health Care Disparities OR Health Care Disparity OR disadvantage* OR Disability OR Disabilities OR “serious mental illness” OR Migrant* OR Immigrant* OR “low income” OR Unemployment OR Unemploy* OR precarious OR homeless OR elderly OR “older adults” OR "chronic disease" OR “single parent*” OR “ethnic minorities” OR “racial minorities” OR refugee OR Uninsured OR pregnant OR child*)	637,324
#2	MH COVID-19 OR TI (COVID-19 OR COVID19 OR Coronavirus OR “Novel coronavirus” OR 2019-nCoV OR SARS-CoV-2 OR SARS2 OR SARS-CoV OR “COVID 19” OR “COVID-19 Virus Disease*” OR “COVID 19 Virus Disease*” OR “COVID-19 Virus Infection*” OR “COVID 19 Virus Infection*” OR “2019-nCoV Infection*” OR “2019 nCoV Infection*” OR “Coronavirus Disease-19” OR “Coronavirus Disease 19” OR “2019 Novel Coronavirus Disease” OR “2019 Novel Coronavirus Infection” OR “2019-nCoV Disease*” OR “2019 nCoV Disease” OR COVID19 OR “Coronavirus Disease 2019” OR “SARS Coronavirus 2 Infection” OR “SARS-CoV-2 Infection*” OR “SARS CoV 2 Infection” OR “COVID-19 Pandemic*” OR “COVID 19 Pandemic”) OR AB (COVID-19 OR COVID19 OR Coronavirus OR “Novel coronavirus” OR 2019-nCoV OR SARS-CoV-2 OR SARS2 OR SARS-CoV OR “COVID 19” OR “COVID-19 Virus Disease*” OR “COVID 19 Virus Disease*” OR “COVID-19 Virus Infection*” OR “COVID 19 Virus Infection*” OR “2019-nCoV Infection*” OR “2019 nCoV Infection*” OR “Coronavirus Disease-19” OR “Coronavirus Disease 19” OR “2019 Novel Coronavirus Disease” OR “2019 Novel Coronavirus Infection” OR “2019-nCoV Disease*” OR “2019 nCoV Disease” OR COVID19 OR “Coronavirus Disease 2019” OR “SARS Coronavirus 2 Infection” OR “SARS-CoV-2 Infection*” OR “SARS CoV 2 Infection” OR “COVID-19 Pandemic*” OR “COVID 19 Pandemic”)	11,635
#3	MH ("Mental health" OR Depression OR Anxiety OR “Stress Disorders, Post-Traumatic” OR “Stress, Psychological” OR Sleep OR “Sleep Disorders, Circadian Rhythm”) OR TI (“Mental health” OR “Psychological health” OR “Psychological impact*” OR “Mental Hygiene" OR Depression* OR depress* OR “Emotional Depression*” OR Anxiety OR Angst OR Nervousness OR Hypervigilance OR Anxiousness OR “Social Anxiety” OR “Social Anxieties” OR PTSD OR PTSS OR “Post-traumatic stress disorder*” OR “Post-Traumatic stress symptoms” OR “Post-Traumatic Neuroses” OR “Post Traumatic Neuroses” OR PTSD OR “Posttraumatic Neuroses” OR “Post-Traumatic Stress Disorder*” OR “Post Traumatic Stress Disorder*” OR “Posttraumatic Stress Disorder*” OR Stress OR “Psychological Stress*” OR “Life Stress*" OR “Psychologic Stress*” OR “Psychological Stressor*” OR sleep* OR insomnia* OR wakeful* OR dyssomn* OR	178,740

	“Sleeping Habit*” OR “Sleep Habit*” OR “Sleep Wake Disorder*” OR “Sleep Disorder*”) OR AB (“Mental health” OR “Psychological health” OR “Psychological impact*” OR “Mental Hygiene” OR Depression* OR depress* OR “Emotional Depression*” OR Anxiety OR Angst OR Nervousness OR Hypervigilance OR Anxiousness OR “Social Anxiety” OR “Social Anxieties” OR PTSD OR PTSS OR “Post-traumatic stress disorder*” OR “Post-Traumatic stress symptoms” OR “Post-Traumatic Neuroses” OR “Post Traumatic Neuroses” OR PTSD OR “Posttraumatic Neuroses” OR “Post-Traumatic Stress Disorder*” OR “Post Traumatic Stress Disorder*” OR “Posttraumatic Stress Disorder*” OR Stress OR “Psychological Stress*” OR “Life Stress*” OR “Psychologic Stress*” OR “Psychological Stressor*” OR sleep* OR insomnia* OR wakeful* OR dyssomn* OR “Sleeping Habit*” OR “Sleep Habit*” OR “Sleep Wake Disorder*” OR “Sleep Disorder*”)	
#4	MH animals NOT human	26,805
#5	#1 AND #2 AND #3 NOT #4	**338**

Supplement 3. Excluded studies after full-text review

1. Not meeting the participant criteria

1. Alves J, Yunker AG, DeFendis A, Xiang AH, Page KA. Children's Anxiety and Physical Activity during COVID-19 in Relation to Prenatal Exposure to Gestational Diabetes. medRxiv. 2020.

2. Durankuş F, Aksu E. Effects of the COVID-19 pandemic on anxiety and depressive symptoms in pregnant women: a preliminary study. J Matern Fetal Neonatal Med. 2020:1-7.

3. García-Fernández L, Romero-Ferreiro V, Padilla S, López-Roldán PD, Monzó-García M, Rodriguez-Jimenez R. The impact on mental health patients of COVID-19 outbreak in Spain. Journal of Psychiatric Research. 2021;136:127-31.

4. Murata S, Rezeppa T, Thoma B, Marengo L, Krancevich K, Chiyka E, et al. The psychiatric sequelae of the COVID-19 pandemic in adolescents, adults, and health care workers. Depression and Anxiety. 2020.

5. Pan SW, Shen GC, Liu C, Hsi JH. Coronavirus stigmatization and psychological distress among Asians in the United States. Ethn Health. 2021;26(1):110-25.

6. Rodriguez-Seijas C, Fields EC, Bottary R, Kark SM, Goldstein MR, Kensinger EA, et al. Comparing the Impact of COVID-19-Related Social Distancing on Mood and Psychiatric Indicators in Sexual and Gender Minority (SGM) and Non-SGM Individuals. Front Psychiatry. 2020;11:590318.

7. Rothe J, Buse J, Uhlmann A, Bluschke A, Roessner V. Changes in emotions and worries during the Covid-19 pandemic: an online-survey with children and adults with and without mental health conditions. Child Adolesc Psychiatry Ment Health. 2021;15(1):11.

8. Schutzwohl M, Mergel E, Schutzwohl M. Social Participation, Inclusion and Mental Well-Being following SARS-CoV-2 Related Restrictions on Going Out: A Follow-up Study from Germany. Psychiatrische Praxis. 2020;47(6):308-18.

9. Seifert J, Meissner C, Birkenstock A, Bleich S, Toto S, Ihlefeld C, et al. Peripandemic psychiatric emergencies: impact of the COVID-19 pandemic on patients according to diagnostic subgroup. Eur Arch Psychiatry Clin Neurosci. 2021:1-12.

10. Singh P, Cumberland WG, Ugarte D, Bruckner TA, Young SD. Association Between Generalized Anxiety Disorder Scores and Online Activity Among US Adults During the COVID-19 Pandemic: Cross-Sectional Analysis. J Med Internet Res. 2020;22(9):e21490.

11. Sun Q, Qin Q, Basta M, Chen B, Li Y. Psychological reactions and insomnia in adults with mental health disorders during the COVID-19 outbreak. BMC Psychiatry. 2021;21(1):19.

2. Not presenting sufficient outcome data

1. Cenk M, Yilmaz Yegit C, Ergenekon P, Toksoy Aksoy A, Gokdemir Y, Erdem Eralp E, et al. The effect

of COVID-19 pandemic on anxiety levels of children with cystic fibrosis and healthy children. Pediatric Pulmonology. 2020;55(SUPPL 2):287.
2. García-Fernández L, Romero-Ferreiro V, López-Roldán PD, Padilla S, Rodriguez-Jimenez R. Mental Health in Elderly Spanish People in Times of COVID-19 Outbreak. Am J Geriatr Psychiatry. 2020;28(10):1040-
3. Pan KY, Kok AAL, Eikelenboom M, Horsfall M, Jörg F, Luteijn RA, et al. The mental health impact of the COVID-19 pandemic on people with and without depressive, anxiety, or obsessive-compulsive disorders: a longitudinal study of three Dutch case-control cohorts. The Lancet Psychiatry. 2021;8(2):121-9.

3. Not comparative study

1. Dalise S, Tramonti F, Armienti E, Niccolini V, Caniglia Tenaglia M, Chisari C. Psycho-social impact of social distancing and isolation due to the COVID-19 containment measures on patients with physical disabilities. European journal of physical and rehabilitation medicine. 2020.
2. Jones DL, Rodriguez VJ, Salazar AS, Montgomerie E, Raccamarich PD, Uribe Starita C, et al. Sex Differences in the Association Between Stress, Loneliness, and COVID-19 Burden among People with HIV in the US. AIDS Res Hum Retroviruses. 2021.

4. Not eligible control group

1. Kitani-Morii F, Kasai T, Horiguchi G, Teramukai S, Ohmichi T, Shinomoto M, et al. Risk factors for neuropsychiatric symptoms in patients with Parkinson's disease during COVID-19 pandemic in Japan. PLoS One. 2021;16(1):e0245864.
2. Ng KYY, Zhou S, Tan SH, Ishak NDB, Goh ZZS, Chua ZY, et al. Understanding the psychological impact of COVID-19 pandemic on patients with cancer, their caregivers, and health care workers in Singapore. JCO Global Oncology. 2020(6):1494-509.
3. Zipprich HM, Teschner U, Witte OW, Schönenberg A, Prell T. Knowledge, attitudes, practices, and burden during the covid-19 pandemic in people with Parkinson's disease in Germany. Journal of Clinical Medicine. 2020;9(6).

5. Results unrelated to the primary or secondary outcomes

1. Ehrler M, Werninger I, Schnider B, Eichelberger DA, Naef N, Disselhoff V, et al. Impact of the COVID-19 pandemic on children with and without risk for neurodevelopmental impairments. Acta Paediatrica, International Journal of Paediatrics. 2021.
2. Nonweiler J, Rattray F, Baulcomb J, Happé F, Absoud M. Prevalence and Associated Factors of

Emotional and Behavioural Difficulties during COVID-19 Pandemic in Children with Neurodevelopmental Disorders. Children (Basel). 2020;7(9).

3. Zgoura P, Seibert FS, Waldecker C, Doevelaar A, Bauer F, Rohn B, et al. Psychological Responses to the Coronavirus Disease 2019 Pandemic in Renal Transplant Recipients. Transplantation Proceedings. 2020;52(9):2671-5.

6. Data measured before the declaration of the pandemic

1. Mergel E, Schützwohl M. A longitudinal study on the COVID-19 pandemic and its divergent effects on social participation and mental health across different study groups with and without mental disorders. Social psychiatry and psychiatric epidemiology. 2021.

2. Ng E, Zhang H. The mental health of immigrants and refugees: Canadian evidence from a nationally linked database. Health Rep. 2020;31(8):3-12.

7. Double publication

1. Santo FD, González-Blanco L, García-Álvarez L, la Fuente-Tomás LD, Moya-Lacasa C, Paniagua G, et al. P.859COVID-19 lockdown in people with severe mental disorders in Spain: do they have a specific psychological reaction? European Neuropsychopharmacology. 2020;40:S475-S6.

8. No details (conference abstract)

1. Van De Poll-franse LV, de Rooij B, Horevoorts N, May AM, Vink G, Koopman M, et al. The impact of the COVID-19 crisis on perceived changes in care and wellbeing of cancer patients and norm participants: Results of the PROFILES registry. Annals of Oncology. 2020;31:S997-S8.

9. Unclear assessment tools

1. Solé B, Verdolini N, Amoretti S, Montejo L, Rosa AR, Hogg B, et al. Effects of the COVID-19 pandemic and lockdown in Spain: comparison between community controls and patients with a psychiatric disorder. Preliminary results from the BRIS-MHC STUDY. J Affect Disord. 2021;281:13-23.

2. Verdolini N, Amoretti S, Montejo L, García-Rizo C, Hogg B, Mezquida G, et al. Resilience and mental health during the COVID-19 pandemic. Journal of Affective Disorders. 2021;283:156-64

10. Written in Chinese

1. Wu J, Yan CJ, Yang YQ, Ye BF, Niu J, Meng XY, et al. Epidemic period of COVID-19: A comparative study of psychological status between breast cancer patients and normal crowd. Chinese Journal of Cancer Prevention and Treatment. 2020;27(18):1508-14.

11. Unavailable full text

1. Nischk D, Voss M. Psychological Distress during the Corona Shutdown: Associations between Behavioral Adaption and Psychological Distress in Individuals with Preexisting Psychiatric Conditions and Healthy Controls. Fortschritte der Neurologie Psychiatrie. 2021.

2. Sabath E, Morales-Montes E. Mental health status during the COVID-19 pandemic of hemodialysis patients. Journal of the American Society of Nephrology. 2020;31:273.

Supplement 4. The results of meta-analysis in this review

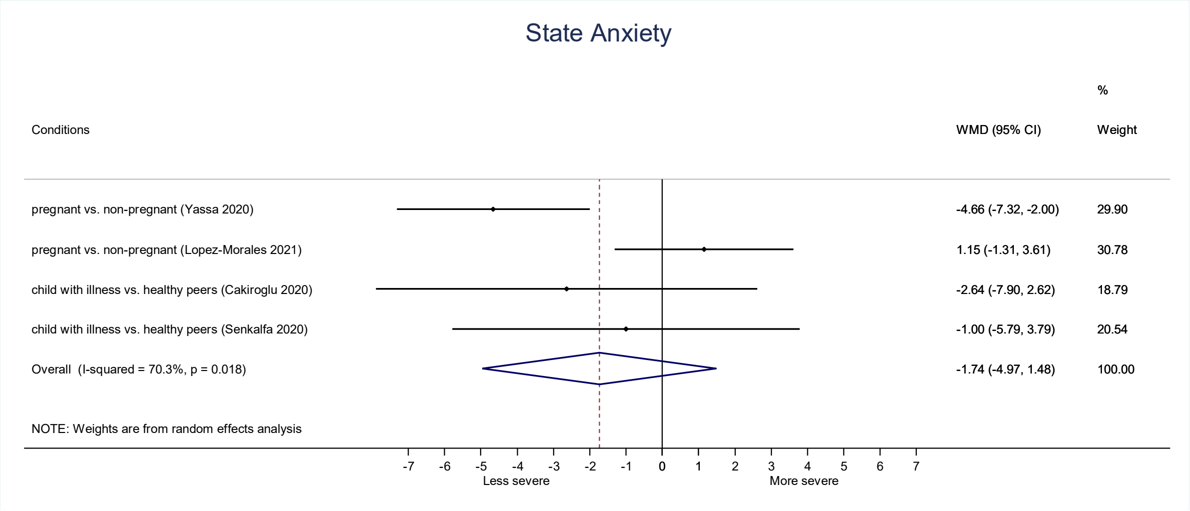

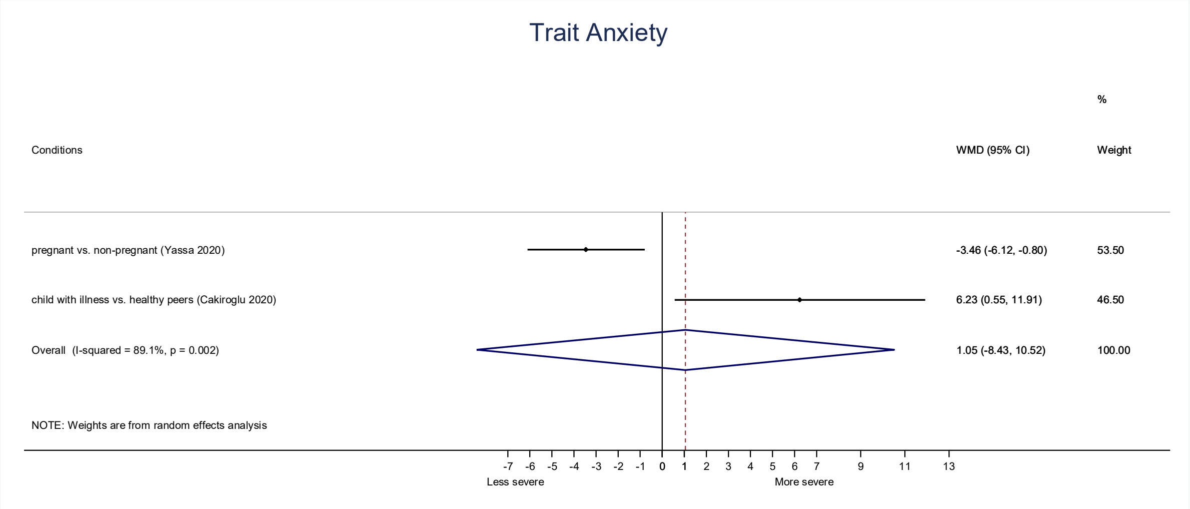

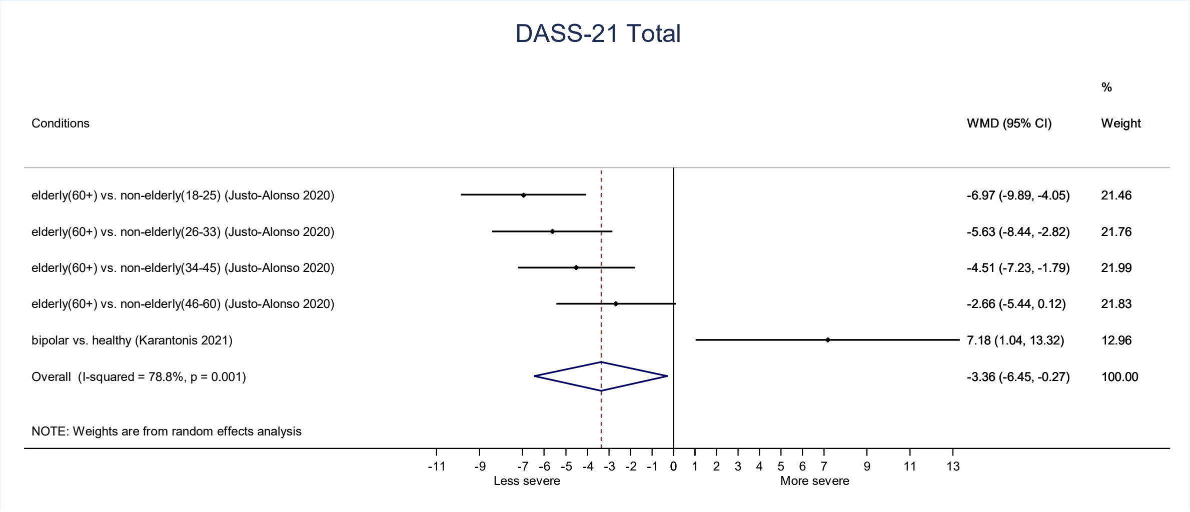

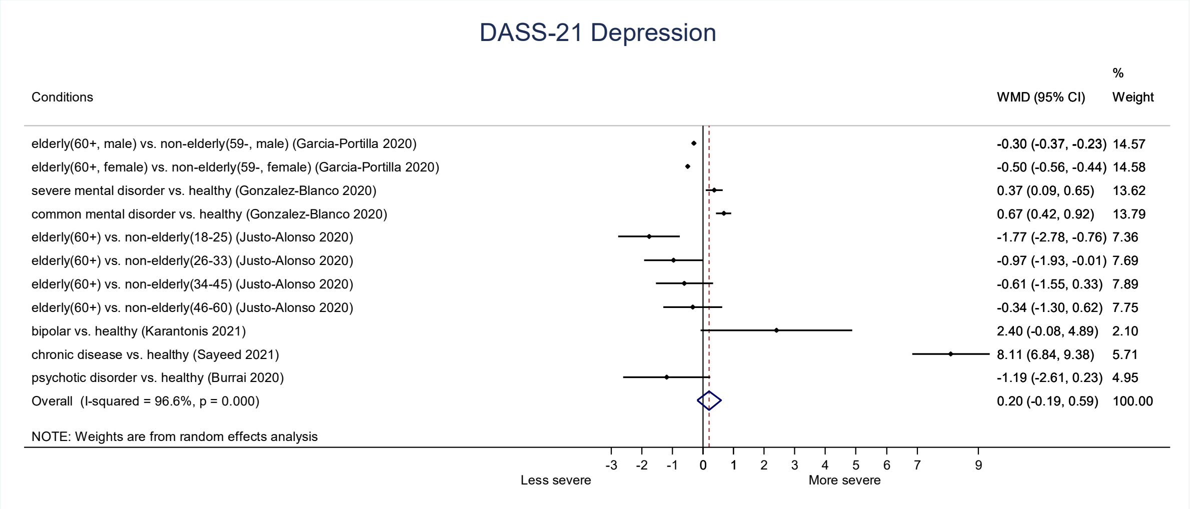
DASS-21 Depression
Conditions
WMD (95% CI)
% Weight
elderly(60+, male) vs. non-elderly(59-, male) (Garcia-Portilla 2020) -0.30 (-0.37, -0.23) 14.57
elderly(60+, female) vs. non-elderly(59-, female) (Garcia-Portilla 2020) -0.50 (-0.56, -0.44) 14.58
severe mental disorder vs. healthy (Gonzalez-Blanco 2020) 0.37 (0.09, 0.65) 13.62
common mental disorder vs. healthy (Gonzalez-Blanco 2020) 0.67 (0.42, 0.92) 13.79
elderly(60+) vs. non-elderly(18-25) (Justo-Alonso 2020) -1.77 (-2.78, -0.76) 7.36
elderly(60+) vs. non-elderly(26-33) (Justo-Alonso 2020) -0.97 (-1.93, -0.01) 7.69
elderly(60+) vs. non-elderly(34-45) (Justo-Alonso 2020) -0.61 (-1.55, 0.33) 7.89
elderly(60+) vs. non-elderly(46-60) (Justo-Alonso 2020) -0.34 (-1.30, 0.62) 7.75
bipolar vs. healthy (Karantonis 2021) 2.40 (-0.08, 4.89) 2.10
chronic disease vs. healthy (Sayeed 2021) 8.11 (6.84, 9.38) 5.71
psychotic disorder vs. healthy (Burrai 2020) -1.19 (-2.61, 0.23) 4.95
Overall (I-squared = 96.6%, p = 0.000) 0.20 (-0.19, 0.59) 100.00
NOTE: Weights are from random effects analysis
-3 -2 -1 0 1 2 3 4 5 6 7 9
Less severe
More severe

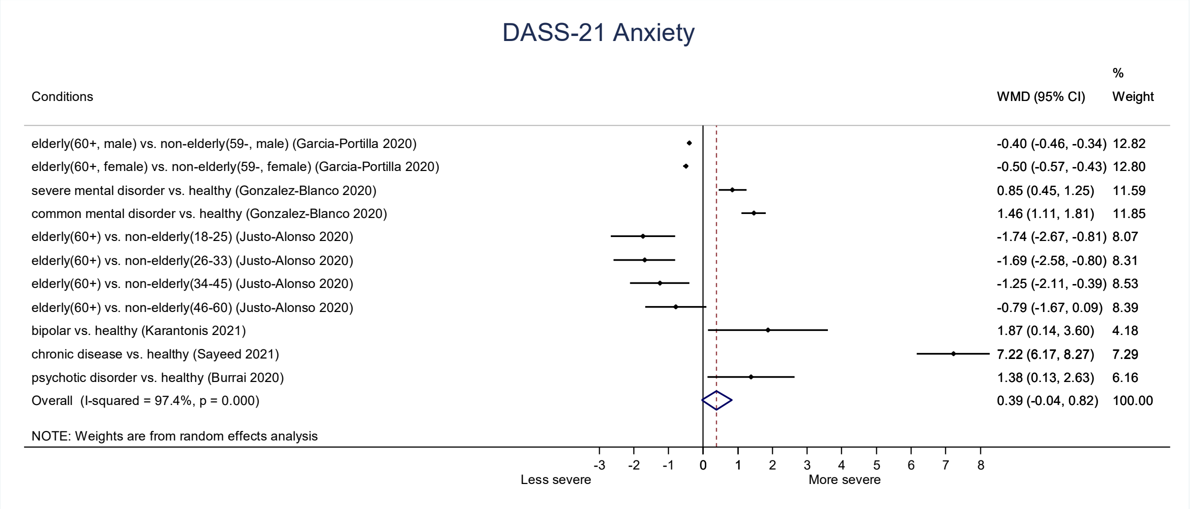
DASS-21 Anxiety
Conditions
WMD (95% CI)
% Weight
elderly(60+, male) vs. non-elderly(59-, male) (Garcia-Portilla 2020) -0.40 (-0.46, -0.34) 12.82
elderly(60+, female) vs. non-elderly(59-, female) (Garcia-Portilla 2020) -0.50 (-0.57, -0.43) 12.80
severe mental disorder vs. healthy (Gonzalez-Blanco 2020) 0.85 (0.45, 1.25) 11.59
common mental disorder vs. healthy (Gonzalez-Blanco 2020) 1.46 (1.11, 1.81) 11.85
elderly(60+) vs. non-elderly(18-25) (Justo-Alonso 2020) -1.74 (-2.67, -0.81) 8.07
elderly(60+) vs. non-elderly(26-33) (Justo-Alonso 2020) -1.69 (-2.58, -0.80) 8.31
elderly(60+) vs. non-elderly(34-45) (Justo-Alonso 2020) -1.25 (-2.11, -0.39) 8.53
elderly(60+) vs. non-elderly(46-60) (Justo-Alonso 2020) -0.79 (-1.67, 0.09) 8.39
bipolar vs. healthy (Karantonis 2021) 1.87 (0.14, 3.60) 4.18
chronic disease vs. healthy (Sayeed 2021) 7.22 (6.17, 8.27) 7.29
psychotic disorder vs. healthy (Burrai 2020) 1.38 (0.13, 2.63) 6.16
Overall (I-squared = 97.4%, p = 0.000) 0.39 (-0.04, 0.82) 100.00
NOTE: Weights are from random effects analysis
-3 -2 -1 0 1 2 3 4 5 6 7 8
Less severe
More severe

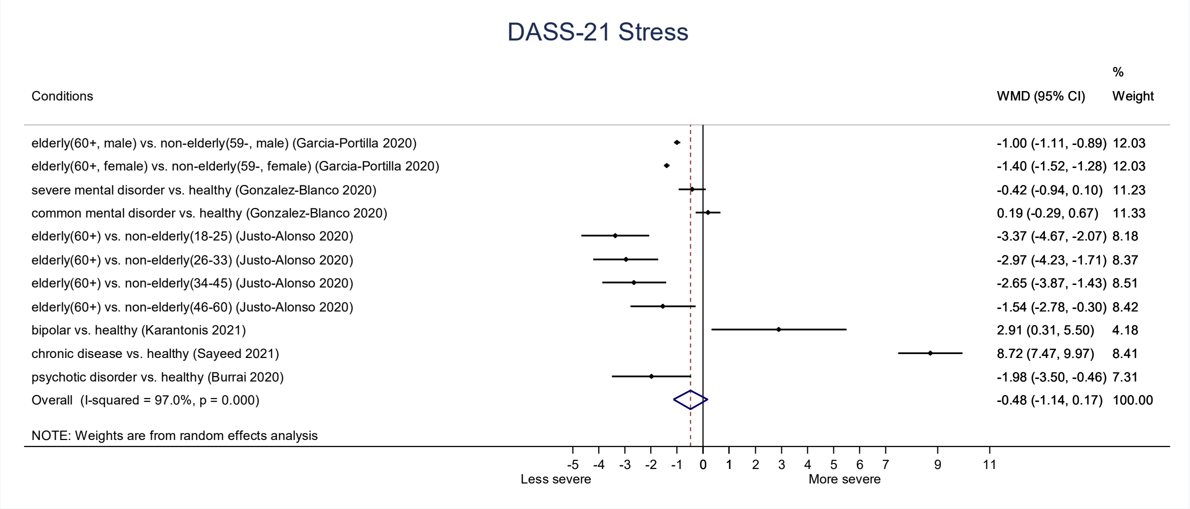
DASS-21 Stress
Conditions
WMD (95% CI)
% Weight
elderly(60+, male) vs. non-elderly(59-, male) (Garcia-Portilla 2020) -1.00 (-1.11, -0.89) 12.03
elderly(60+, female) vs. non-elderly(59-, female) (Garcia-Portilla 2020) -1.40 (-1.52, -1.28) 12.03
severe mental disorder vs. healthy (Gonzalez-Blanco 2020) -0.42 (-0.94, 0.10) 11.23
common mental disorder vs. healthy (Gonzalez-Blanco 2020) 0.19 (-0.29, 0.67) 11.33
elderly(60+) vs. non-elderly(18-25) (Justo-Alonso 2020) -3.37 (-4.67, -2.07) 8.18
elderly(60+) vs. non-elderly(26-33) (Justo-Alonso 2020) -2.97 (-4.23, -1.71) 8.37
elderly(60+) vs. non-elderly(34-45) (Justo-Alonso 2020) -2.65 (-3.87, -1.43) 8.51
elderly(60+) vs. non-elderly(46-60) (Justo-Alonso 2020) -1.54 (-2.78, -0.30) 8.42
bipolar vs. healthy (Karantonis 2021) 2.91 (0.31, 5.50) 4.18
chronic disease vs. healthy (Sayeed 2021) 8.72 (7.47, 9.97) 8.41
psychotic disorder vs. healthy (Burrai 2020) -1.98 (-3.50, -0.46) 7.31
Overall (I-squared = 97.0%, p = 0.000) -0.48 (-1.14, 0.17) 100.00
NOTE: Weights are from random effects analysis
-5 -4 -3 -2 -1 0 1 2 3 4 5 6 7 9 11
Less severe
More severe

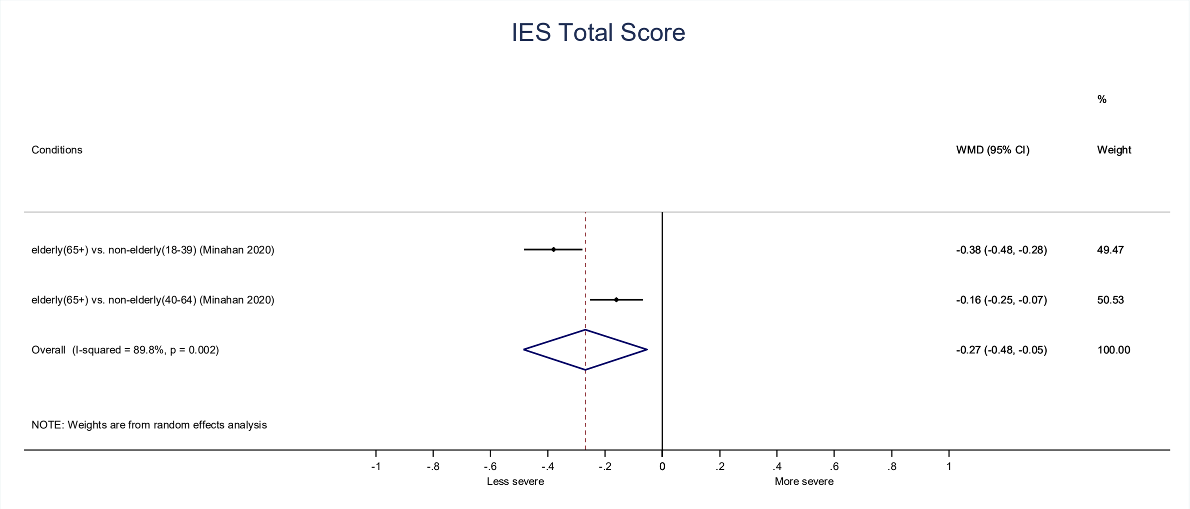
IES Total Score
Conditions
WMD (95% CI)
%
Weight
elderly(65+) vs. non-elderly(18-39) (Minahan 2020)
-0.38 (-0.48, -0.28)
49.47
elderly(65+) vs. non-elderly(40-64) (Minahan 2020)
-0.16 (-0.25, -0.07)
50.53
Overall (I-squared = 89.8%, p = 0.002)
-0.27 (-0.48, -0.05)
100.00
NOTE: Weights are from random effects analysis
-1 -.8 -.6 -.4 -.2 0 .2 .4 .6 .8 1
Less severe
More severe

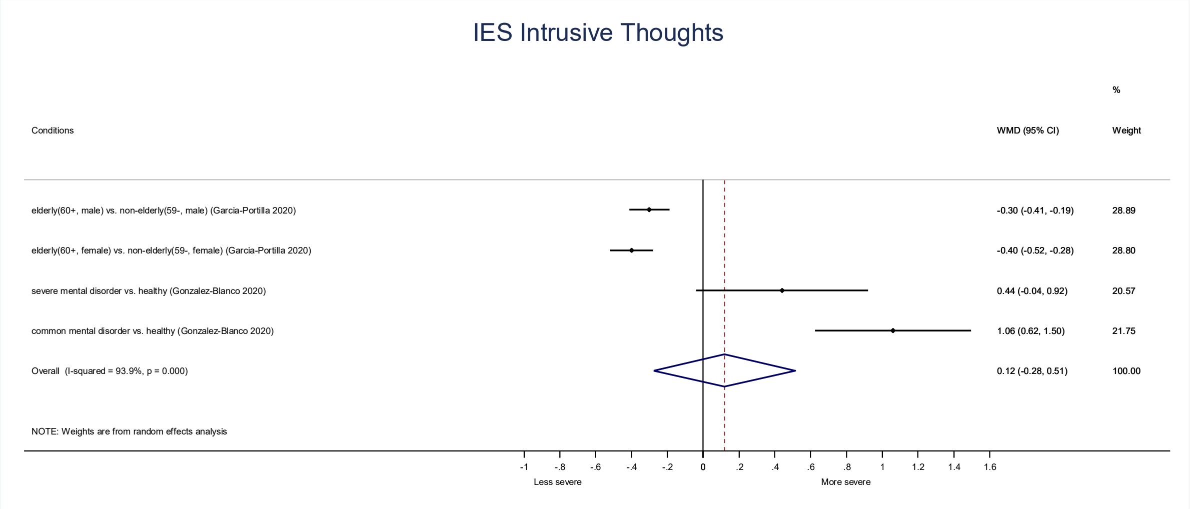
IES Intrusive Thoughts
Conditions
WMD (95% CI)
%
Weight
elderly(60+, male) vs. non-elderly(59-, male) (Garcia-Portilla 2020)
-0.30 (-0.41, -0.19)
28.89
elderly(60+, female) vs. non-elderly(59-, female) (Garcia-Portilla 2020)
-0.40 (-0.52, -0.28)
28.80
severe mental disorder vs. healthy (Gonzalez-Blanco 2020)
0.44 (-0.04, 0.92)
20.57
common mental disorder vs. healthy (Gonzalez-Blanco 2020)
1.06 (0.62, 1.50)
21.75
Overall (I-squared = 93.9%, p = 0.000)
0.12 (-0.28, 0.51)
100.00
NOTE: Weights are from random effects analysis
-1 -.8 -.6 -.4 -.2 0 .2 .4 .6 .8 1 1.2 1.4 1.6
Less severe
More severe

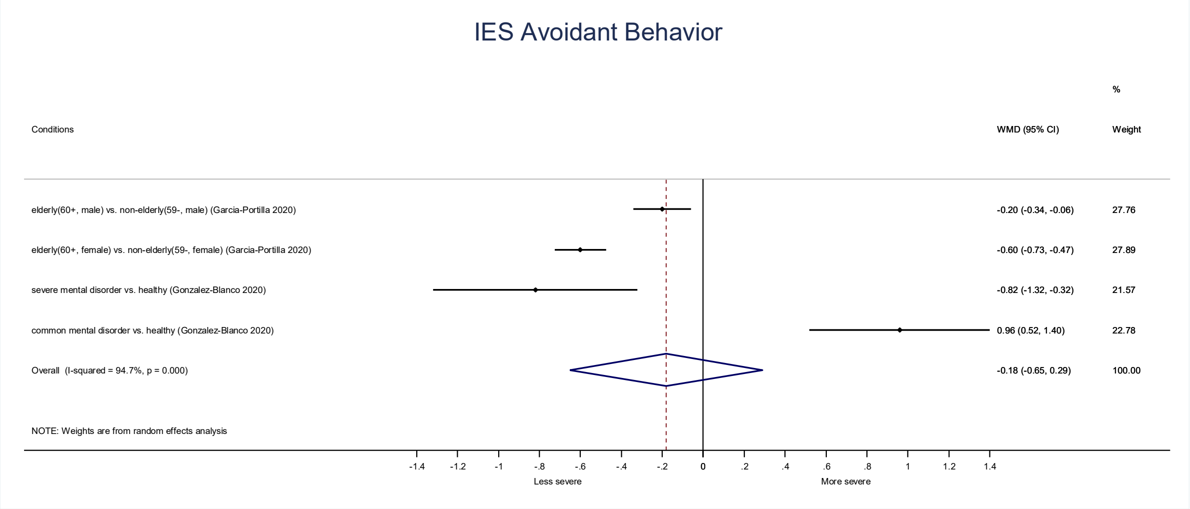
IES Avoidant Behavior
Conditions
WMD (95% CI)
%
Weight
elderly(60+, male) vs. non-elderly(59-, male) (Garcia-Portilla 2020)
-0.20 (-0.34, -0.06)
27.76
elderly(60+, female) vs. non-elderly(59-, female) (Garcia-Portilla 2020)
-0.60 (-0.73, -0.47)
27.89
severe mental disorder vs. healthy (Gonzalez-Blanco 2020)
-0.82 (-1.32, -0.32)
21.57
common mental disorder vs. healthy (Gonzalez-Blanco 2020)
0.96 (0.52, 1.40)
22.78
Overall (I-squared = 94.7%, p = 0.000)
-0.18 (-0.65, 0.29)
100.00
NOTE: Weights are from random effects analysis
-1.4 -1.2 -1 -.8 -.6 -.4 -.2 0 .2 .4 .6 .8 1 1.2 1.4
Less severe
More severe

IES-R Total Score

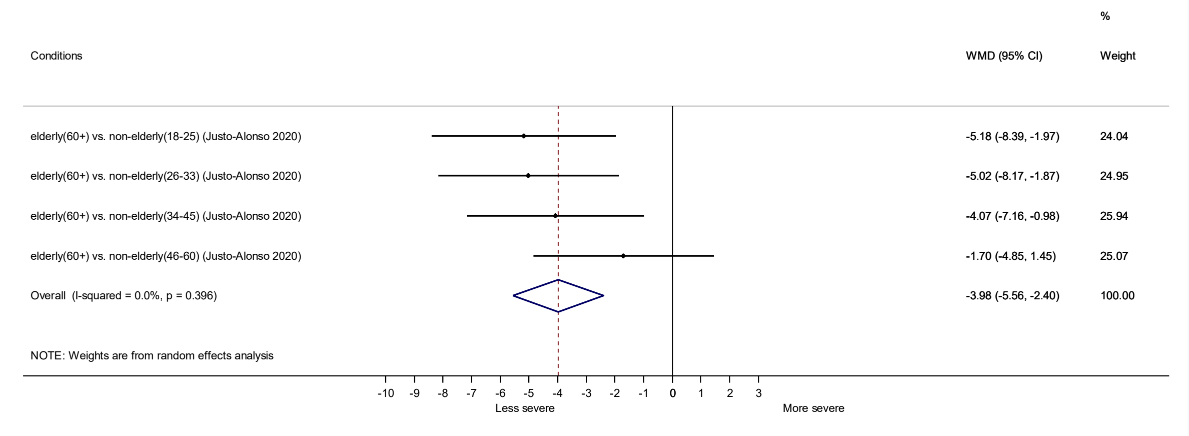

IES-R Hyperactivation

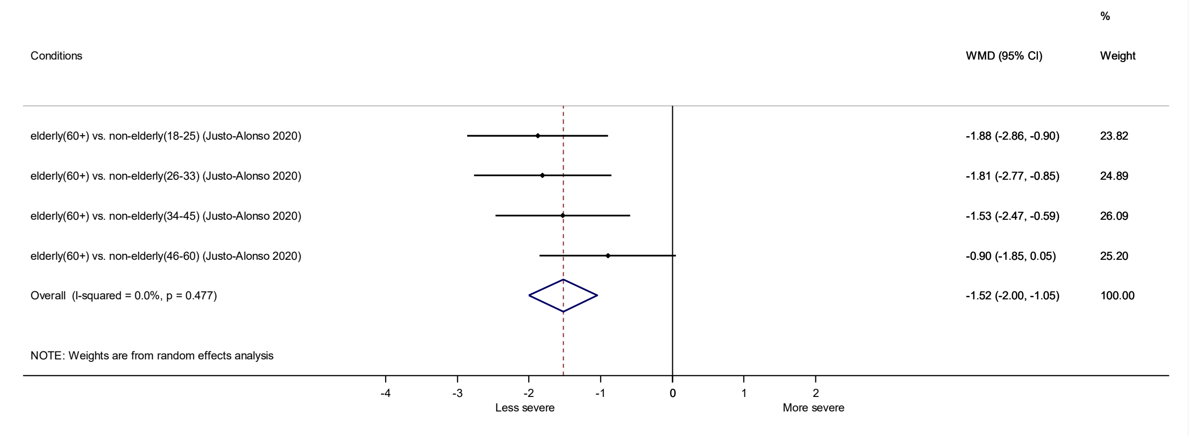

IES-R Evitation

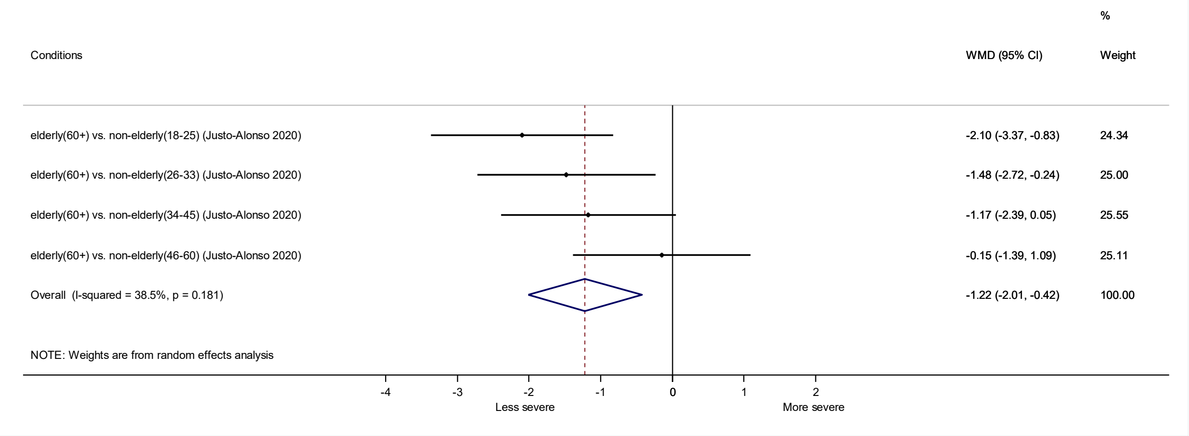

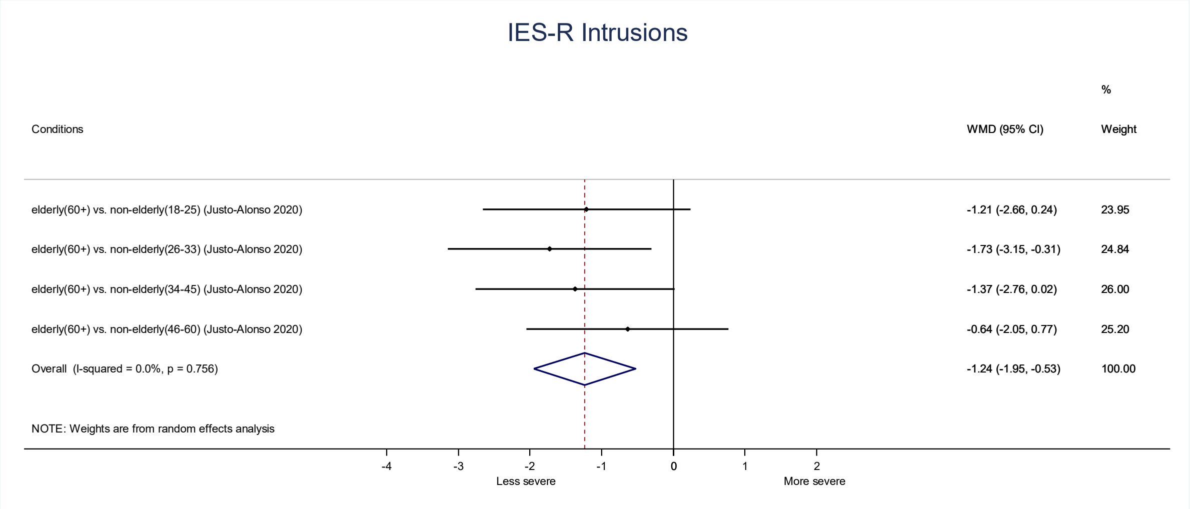
IES-R Intrusions
Conditions
WMD (95% CI)
%
Weight
elderly(60+) vs. non-elderly(18-25) (Justo-Alonso 2020) -1.21 (-2.66, 0.24) 23.95
elderly(60+) vs. non-elderly(26-33) (Justo-Alonso 2020) -1.73 (-3.15, -0.31) 24.84
elderly(60+) vs. non-elderly(34-45) (Justo-Alonso 2020) -1.37 (-2.76, 0.02) 26.00
elderly(60+) vs. non-elderly(46-60) (Justo-Alonso 2020) -0.64 (-2.05, 0.77) 25.20
Overall (I-squared = 0.0%, p = 0.756) -1.24 (-1.95, -0.53) 100.00
NOTE: Weights are from random effects analysis
-4 -3 -2 -1 0 1 2
Less severe
More severe

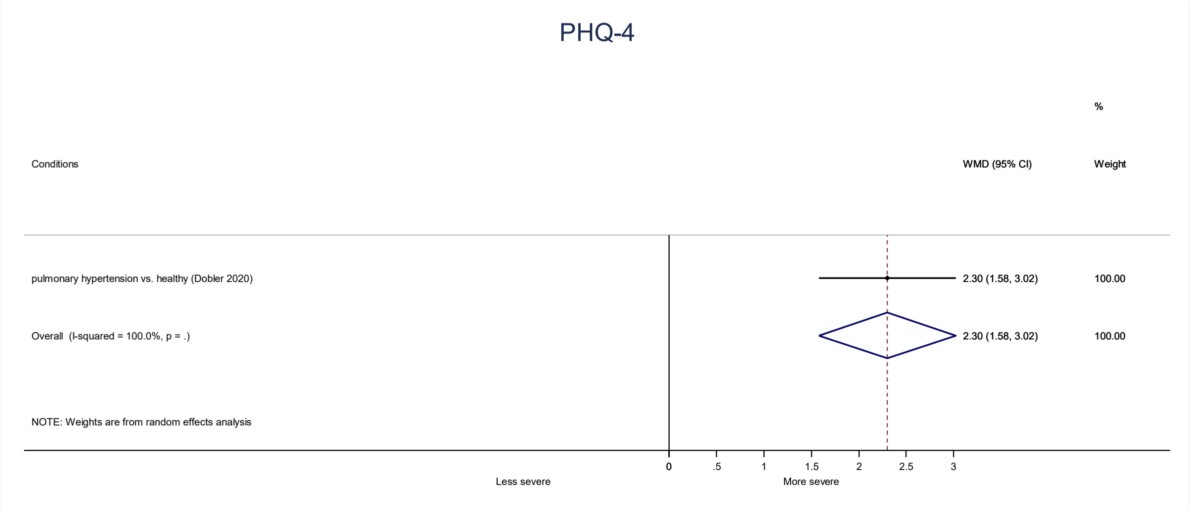
PHQ-4
Conditions
WMD (95% CI)
%
Weight
pulmonary hypertension vs. healthy (Dobler 2020) 2.30 (1.58, 3.02) 100.00
Overall (I-squared = 100.0%, p = .) 2.30 (1.58, 3.02) 100.00
NOTE: Weights are from random effects analysis
0 .5 1 1.5 2 2.5 3
Less severe
More severe

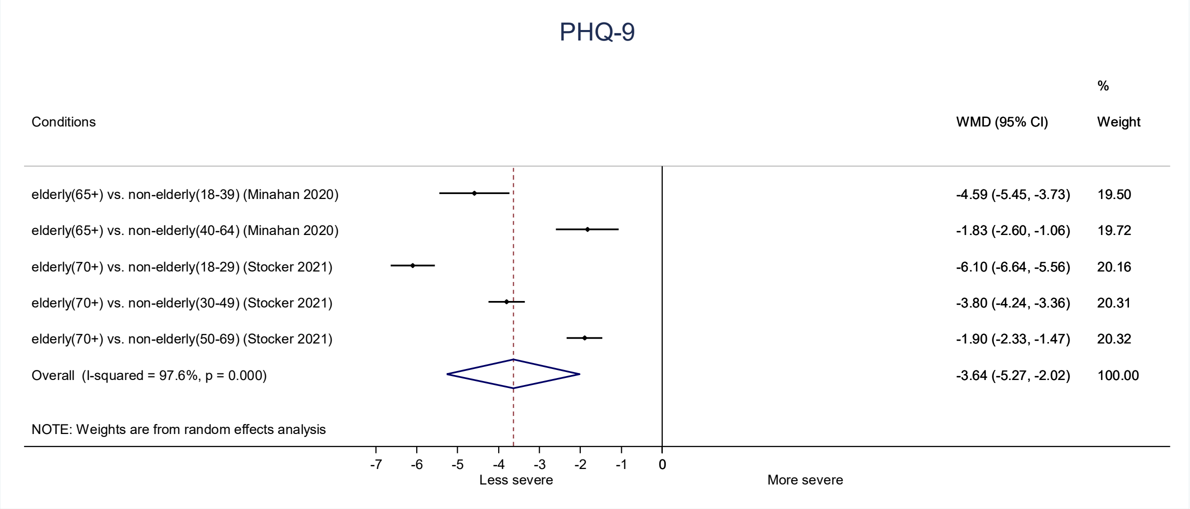
PHQ-9
Conditions
WMD (95% CI)
%
Weight
elderly(65+) vs. non-elderly(18-39) (Minahan 2020) -4.59 (-5.45, -3.73) 19.50
elderly(65+) vs. non-elderly(40-64) (Minahan 2020) -1.83 (-2.60, -1.06) 19.72
elderly(70+) vs. non-elderly(18-29) (Stocker 2021) -6.10 (-6.64, -5.56) 20.16
elderly(70+) vs. non-elderly(30-49) (Stocker 2021) -3.80 (-4.24, -3.36) 20.31
elderly(70+) vs. non-elderly(50-69) (Stocker 2021) -1.90 (-2.33, -1.47) 20.32
Overall (I-squared = 97.6%, p = 0.000) -3.64 (-5.27, -2.02) 100.00
NOTE: Weights are from random effects analysis
-7 -6 -5 -4 -3 -2 -1 0
Less severe
More severe

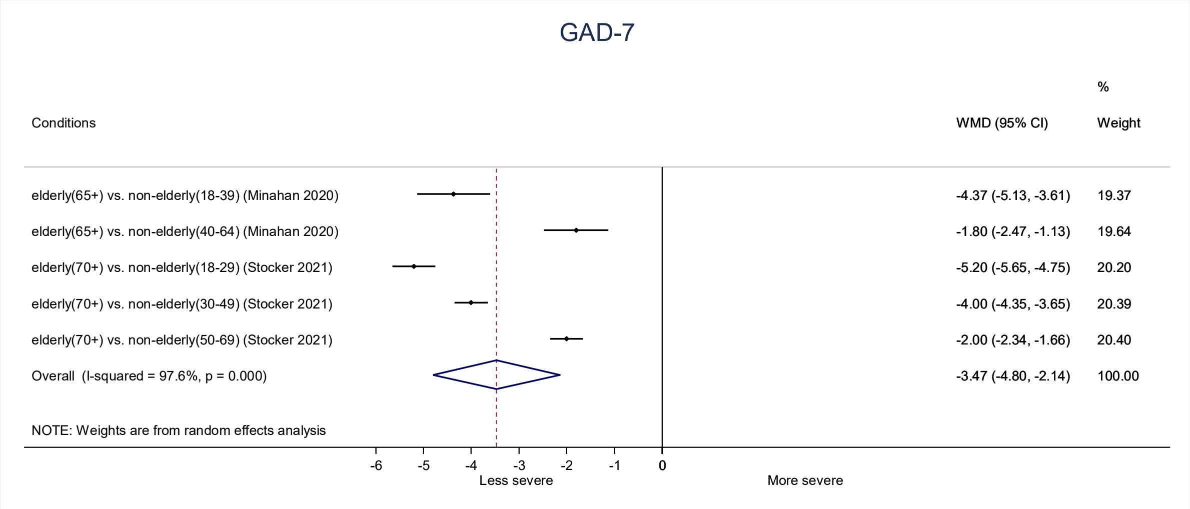
GAD-7
Conditions
WMD (95% CI)
%
Weight
elderly(65+) vs. non-elderly(18-39) (Minahan 2020) -4.37 (-5.13, -3.61) 19.37
elderly(65+) vs. non-elderly(40-64) (Minahan 2020) -1.80 (-2.47, -1.13) 19.64
elderly(70+) vs. non-elderly(18-29) (Stocker 2021) -5.20 (-5.65, -4.75) 20.20
elderly(70+) vs. non-elderly(30-49) (Stocker 2021) -4.00 (-4.35, -3.65) 20.39
elderly(70+) vs. non-elderly(50-69) (Stocker 2021) -2.00 (-2.34, -1.66) 20.40
Overall (I-squared = 97.6%, p = 0.000) -3.47 (-4.80, -2.14) 100.00
NOTE: Weights are from random effects analysis
-6 -5 -4 -3 -2 -1 0
Less severe
More severe

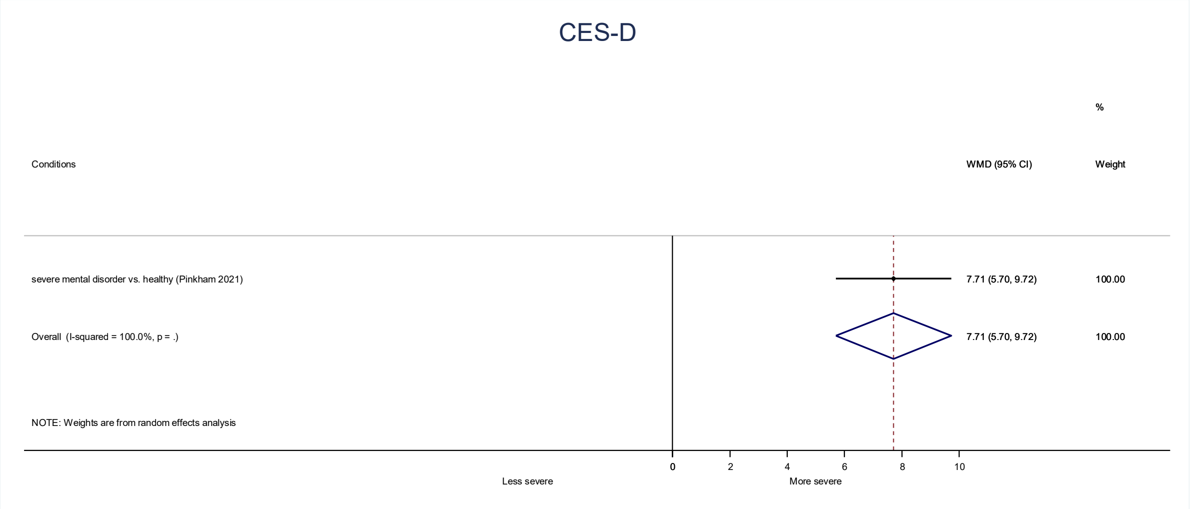
CES-D
Conditions
WMD (95% CI)
%
Weight
severe mental disorder vs. healthy (Pinkham 2021) 7.71 (5.70, 9.72) 100.00
Overall (I-squared = 100.0%, p = .) 7.71 (5.70, 9.72) 100.00
NOTE: Weights are from random effects analysis
0 2 4 6 8 10
Less severe
More severe

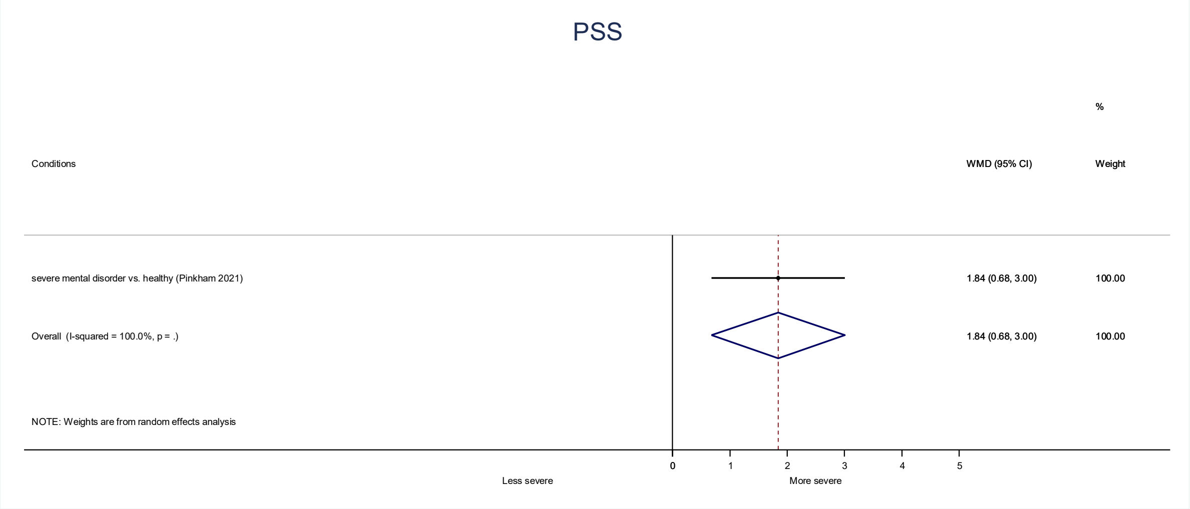
PSS
Conditions
WMD (95% CI)
%
Weight
severe mental disorder vs. healthy (Pinkham 2021) 1.84 (0.68, 3.00) 100.00
Overall (I-squared = 100.0%, p = .) 1.84 (0.68, 3.00) 100.00
NOTE: Weights are from random effects analysis
0 1 2 3 4 5
Less severe
More severe

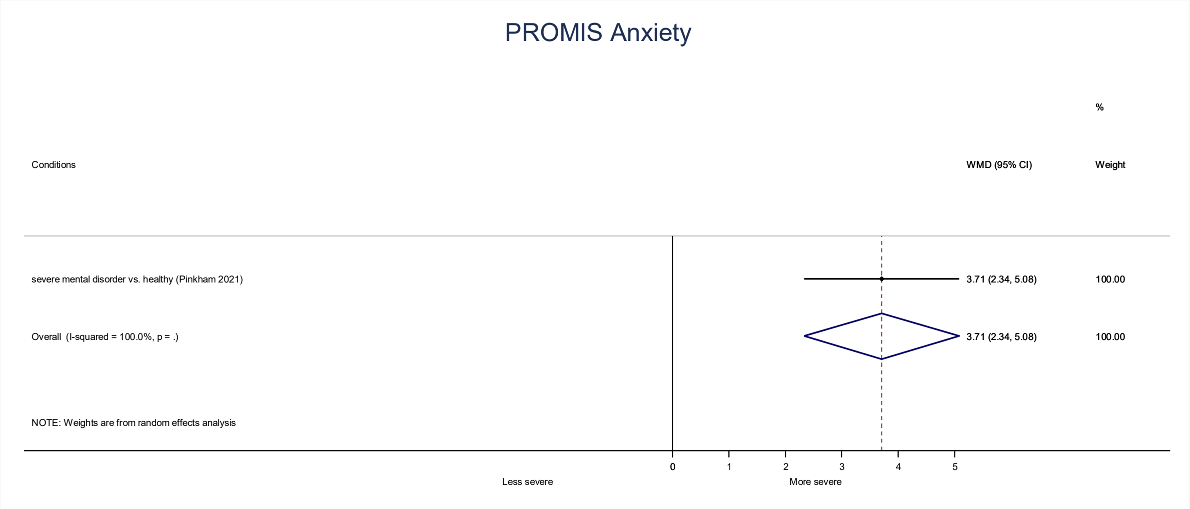
PROMIS Anxiety
Conditions
WMD (95% CI)
%
Weight
severe mental disorder vs. healthy (Pinkham 2021)
3.71 (2.34, 5.08)
100.00
Overall (I-squared = 100.0%, p = .)
3.71 (2.34, 5.08)
100.00
NOTE: Weights are from random effects analysis
0
1
2
3
4
5
Less severe
More severe

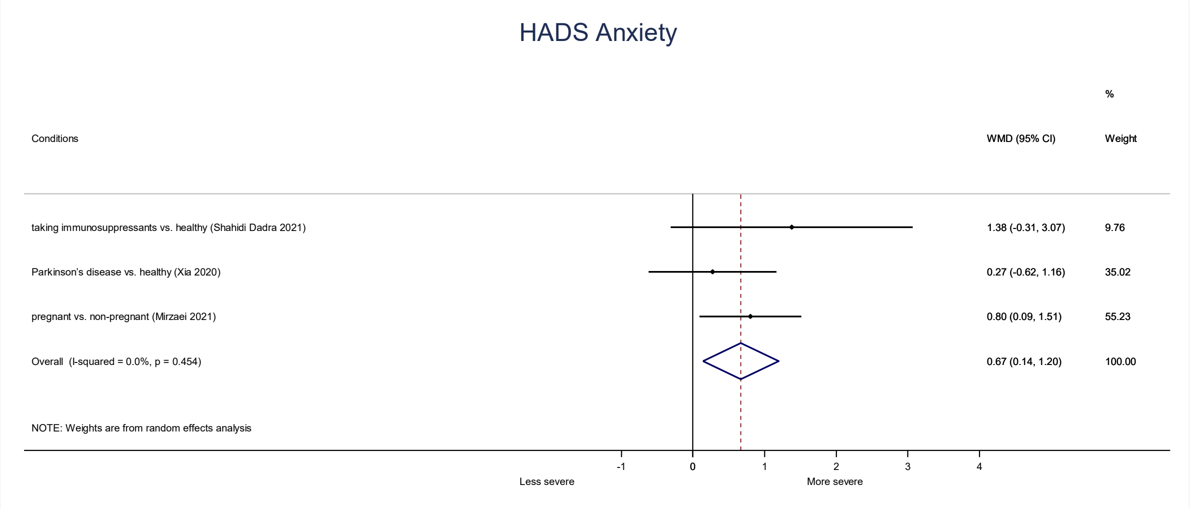
HADS Anxiety
Conditions
WMD (95% CI)
%
Weight
taking immunosuppressants vs. healthy (Shahidi Dadra 2021)
1.38 (-0.31, 3.07)
9.76
Parkinson's disease vs. healthy (Xia 2020)
0.27 (-0.62, 1.16)
35.02
pregnant vs. non-pregnant (Mirzaei 2021)
0.80 (0.09, 1.51)
55.23
Overall (I-squared = 0.0%, p = 0.454)
0.67 (0.14, 1.20)
100.00
NOTE: Weights are from random effects analysis
-1
0
1
2
3
4
Less severe
More severe

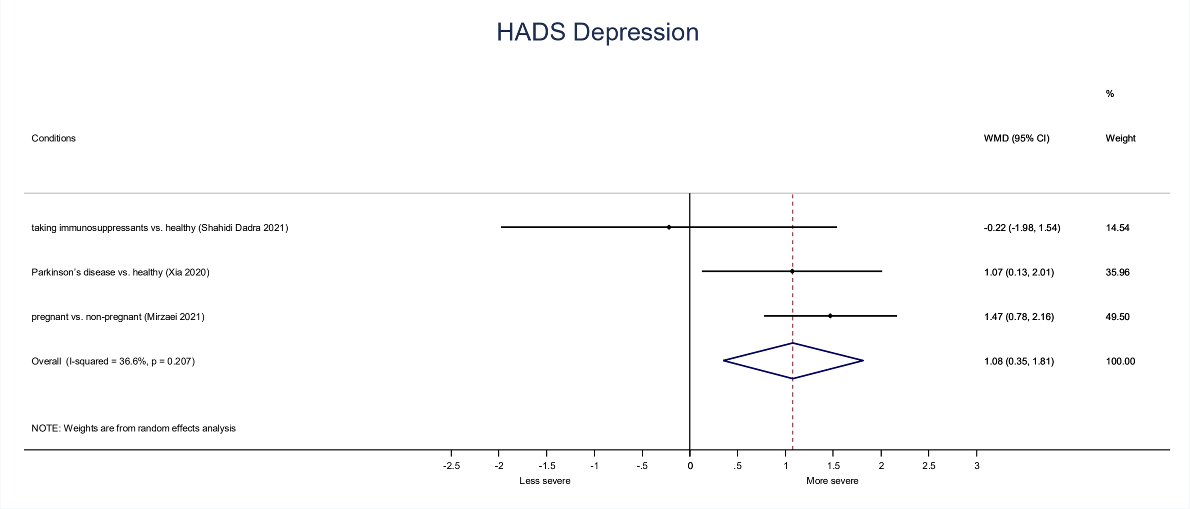
HADS Depression
Conditions
WMD (95% CI)
%
Weight
taking immunosuppressants vs. healthy (Shahidi Dadra 2021)
-0.22 (-1.98, 1.54)
14.54
Parkinson's disease vs. healthy (Xia 2020)
1.07 (0.13, 2.01)
35.96
pregnant vs. non-pregnant (Mirzaei 2021)
1.47 (0.78, 2.16)
49.50
Overall (I-squared = 36.6%, p = 0.207)
1.08 (0.35, 1.81)
100.00
NOTE: Weights are from random effects analysis
-2.5
-2
-1.5
-1
-.5
0
.5
1
1.5
2
2.5
3
Less severe
More severe

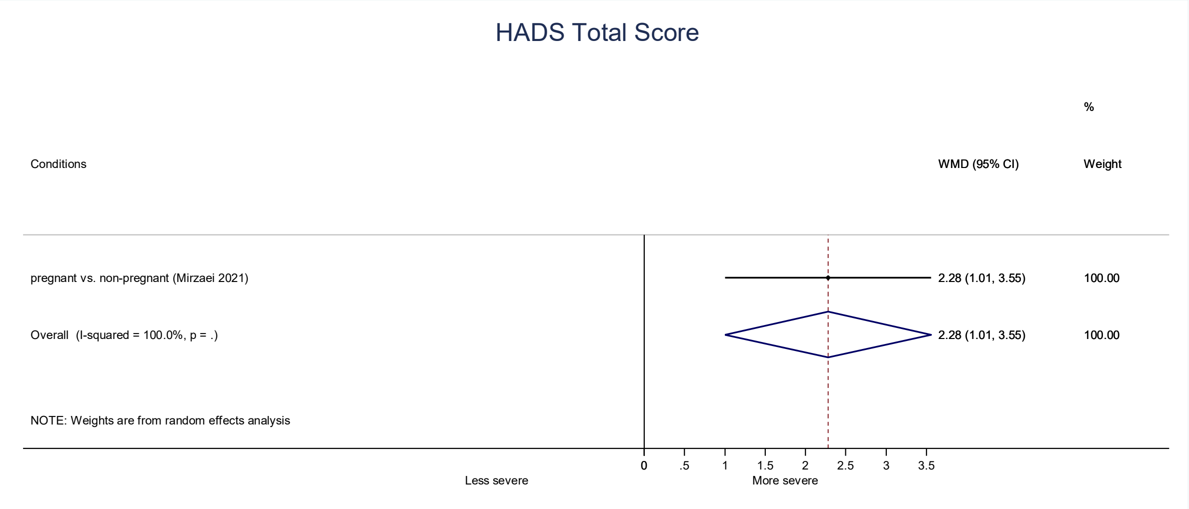
HADS Total Score
%
Conditions
WMD (95% CI)
Weight
pregnant vs. non-pregnant (Mirzaei 2021)
2.28 (1.01, 3.55)
100.00
Overall (I-squared = 100.0%, p = .)
2.28 (1.01, 3.55)
100.00
NOTE: Weights are from random effects analysis
0 .5 1 1.5 2 2.5 3 3.5
Less severe
More severe

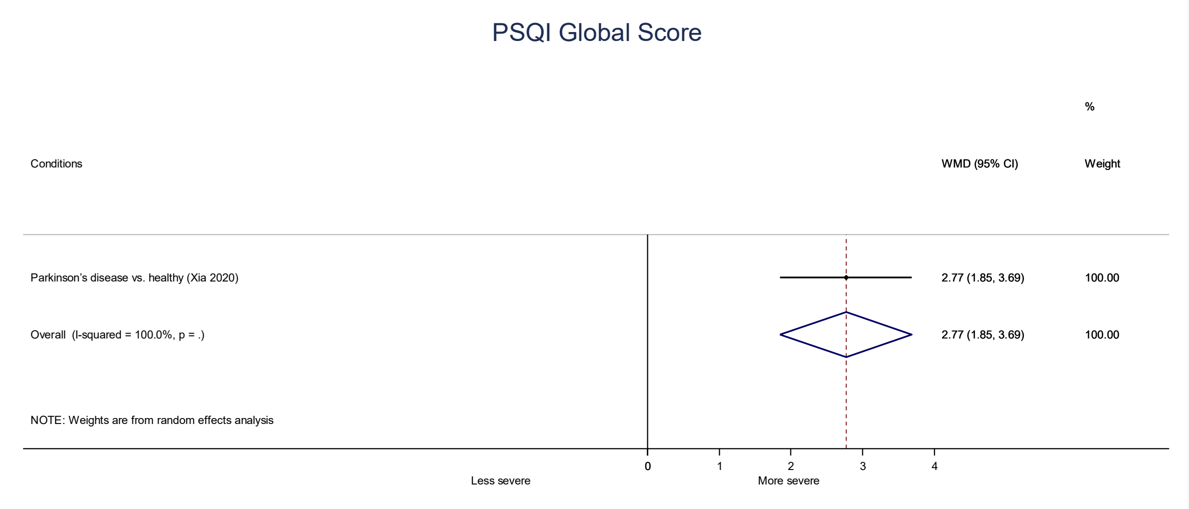
PSQI Global Score
%
Conditions
WMD (95% CI)
Weight
Parkinson's disease vs. healthy (Xia 2020)
2.77 (1.85, 3.69)
100.00
Overall (I-squared = 100.0%, p = .)
2.77 (1.85, 3.69)
100.00
NOTE: Weights are from random effects analysis
0 1 2 3 4
Less severe
More severe

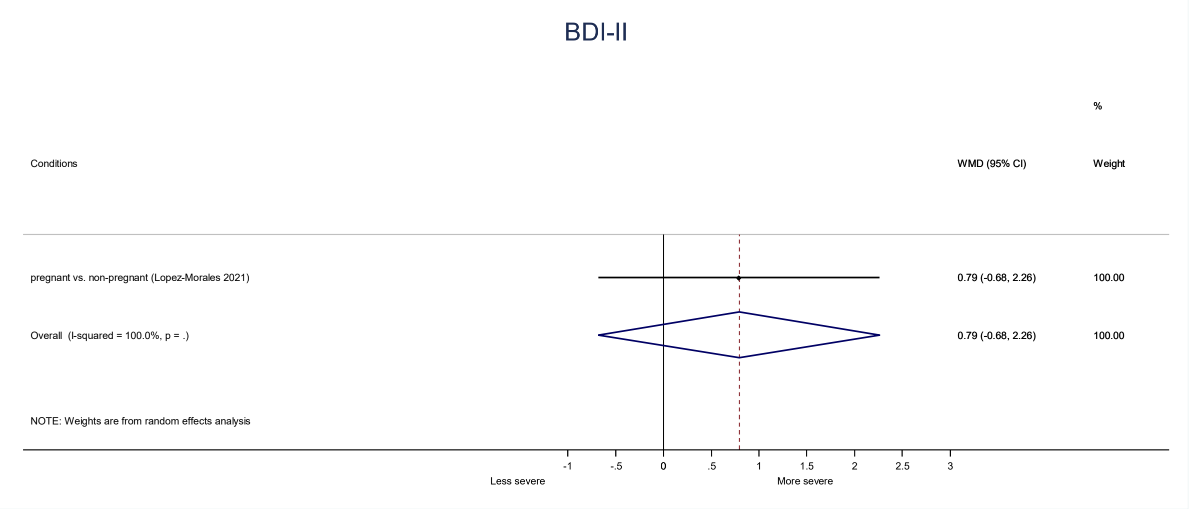
BDI-II
%
Conditions
WMD (95% CI)
Weight
pregnant vs. non-pregnant (Lopez-Morales 2021)
0.79 (-0.68, 2.26)
100.00
Overall (I-squared = 100.0%, p = .)
0.79 (-0.68, 2.26)
100.00
NOTE: Weights are from random effects analysis
-1 -.5 0 .5 1 1.5 2 2.5 3
Less severe
More severe

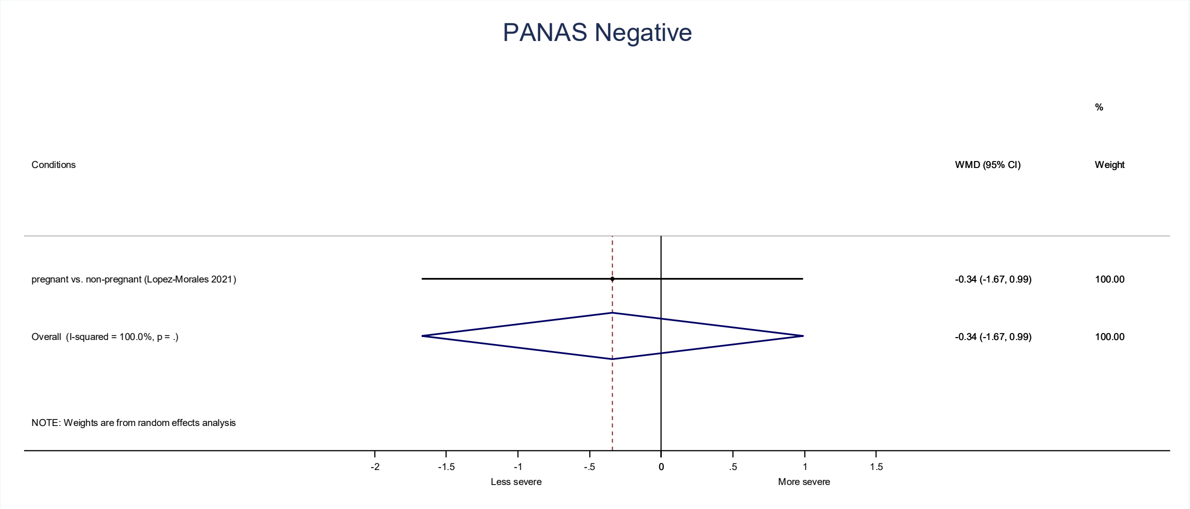
PANAS Negative
%
Conditions
WMD (95% CI)
Weight
pregnant vs. non-pregnant (Lopez-Morales 2021)
-0.34 (-1.67, 0.99)
100.00
Overall (I-squared = 100.0%, p = .)
-0.34 (-1.67, 0.99)
100.00
NOTE: Weights are from random effects analysis
-2
-1.5
-1
-.5
0
.5
1
1.5
Less severe
More severe

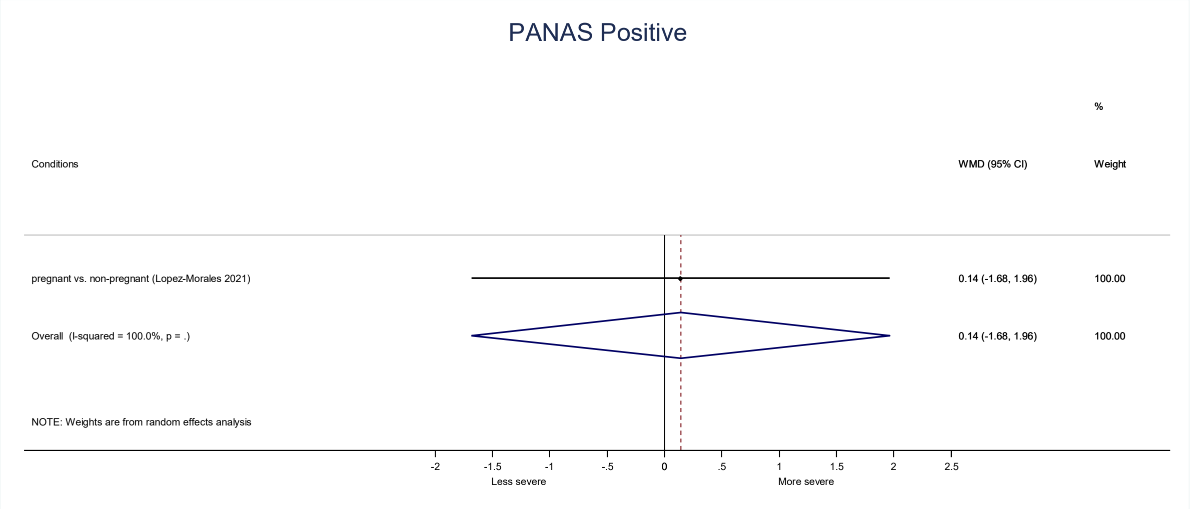
PANAS Positive
%
Conditions
WMD (95% CI)
Weight
pregnant vs. non-pregnant (Lopez-Morales 2021)
0.14 (-1.68, 1.96)
100.00
Overall (I-squared = 100.0%, p = .)
0.14 (-1.68, 1.96)
100.00
NOTE: Weights are from random effects analysis
-2
-1.5
-1
-.5
0
.5
1
1.5
2
2.5
Less severe
More severe
